# Carboxylate and coordination influence on the formation of an active Ru^V^ Oxo species

**DOI:** 10.1038/s41598-025-89062-5

**Published:** 2025-02-18

**Authors:** Jamal El-Abid, Kevin M. Dorst, Andrew K. Inge, Oscar Verho, Varun Kundi, Priyank V. Kumar, Anders Thapper, Biswanath Das

**Affiliations:** 1 Department of Organic Chemistry, Arrhenius Laboratory Stockholm University, Svante Arrhenius Väg 16C, 10691 Stockholm, Sweden; 2 Department of Materials and Environmental Chemistry, Stockholm University, Svante Arrhenius Väg 16C, 106 91 Stockholm, Sweden; 3 Department of Medicinal Chemistry, Biomedicinskt Centrum BMC, Uppsala University, 75123 Uppsala, Sweden; 4 School of Chemical Engineering, The University of New South Wales, Sydney, NSW 2052 Australia; 5 Department of Chemistry–Ångström Laboratory, Uppsala University, P.O. Box 523, 75120 Uppsala, Sweden

**Keywords:** Chemistry, Coordination chemistry

## Abstract

Understanding the structure of Ru(V)-oxo species is crucial for designing novel catalysts for sustainable energy applications, such as water splitting for green hydrogen production. This study reports the EPR detection of a Ru(V)-oxo intermediate stabilized by terpyridine and phenanthroline carboxylate ligands. The interaction between the carboxylate group and the ruthenium center, along with PCET-dependent hemilability under oxidative conditions, plays a critical role in achieving the high-valent state. Subtle changes in the coordination environment around the central metal also proved to be essential. Low-temperature NMR, high-resolution mass spectrometry, UV–Vis spectroscopy, and density functional theory calculations support these findings.

High-valent ruthenium (V) oxo moieties have been suggested to be reactive intermediates in several important chemical processes.^1,2^ This includes water, alcohol, and C-H bond oxidations. Among all of these oxidation reactions, water oxidation has enticed enormous attention as it is the most energy-demanding step on the way to producing green hydrogen from water electrolysis.^3–6^ It comprises multiple synchronized protons and electrons transfer (PCET).^7^

One of the proposed mechanisms of water oxidation (WO) catalyzed by the molecular ruthenium complexes involves the formation of an active Ru^V^(Oxo) unit that undergoes water nucleophilic attack (WNA) and O–O bond formation.^4,8,9^ This step has been identified as the rate-limiting in the process of generating O_2_ from water, rendering Ru^V^(Oxo) intermediates exceptionally intriguing subjects for further investigation. The other commonly accepted mechanism for the crucial O–O bond formation involves a bimolecular (I2M) pathway, requiring two Ru^V^(Oxo) or Ru^IV^(Oxyl) species to approach each other.^4,8,9^ However, the sterically hindered ligand framework favours the WNA mechanism over the I2M pathway. A thorough understanding of how ligand structures influence the formation of high-valent intermediates can provide invaluable insights into the design of more efficient catalysts and electrocatalysts.

The high-valent oxo intermediates can be generated both chemically and electrochemically.^1,10–12^ While the electrochemical method stands out as a preferable and more environmentally friendly approach for water oxidation applications, the chemical route offers the advantage of easily adjusting the equivalence of external oxidants – a crucial factor in enhancing the stability of transient species. An additional significant motivation for exploring high-valent molecular species is to identify a robust ligand environment capable of supporting multiple redox states of the metal centers. Such an environment can facilitate Proton-Coupled Electron Transfer (PCET) reactions, reducing the overall charge of high-valent species and mitigating self-oxidation.^8,13–15^

In this study, we present two well-characterized ruthenium complexes with slightly different ligand environments, [(^tbu^bpy)_2_Ru^II^(phenCO_2_)](PF_6_)·H_2_O (**1**) and [(^tbu^tpy)Ru^II^(phenCO_2_)](PF_6_) (**2**) (Fig. 1), aiming to elucidate the influence of coordination and carboxylate group interaction on the formation of the Ru^V^(Oxo) intermediate under Ce(IV)-induced conditions. EPR, HRMS, and low-temperature NMR spectroscopic techniques were employed to identify the in-situ oxidized species.

**Fig. 1:**
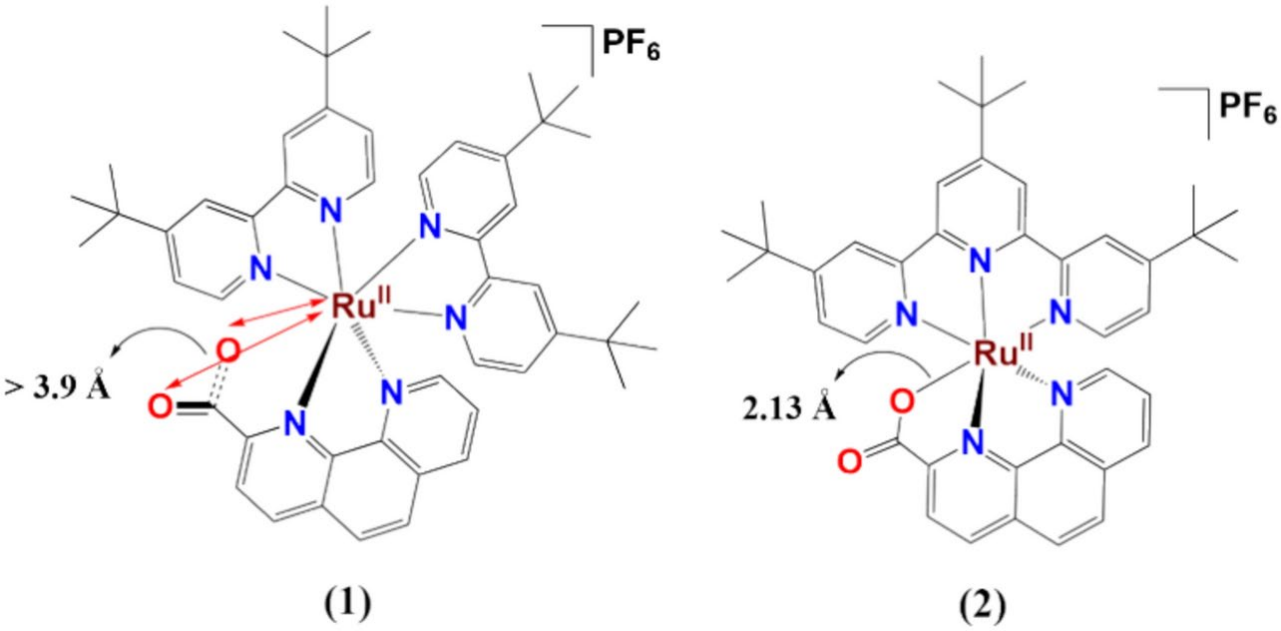
Graphical depiction of the coordination environment around the ruthenium centres in **1** and **2**. The nearest distances between Ru and O atoms are highlighted.

The addition of an excess (10–20 equiv.) of Ce^IV^ as a chemical oxidant generated several new species characterized by EPR, but only **2** generated a Ru^V^-(Oxo) species which shows features compatible with water oxidation, whereas **1** resulted in a Ru^III^ species that is catalytically inactive, and no higher-valent species could be observed.

The new heteroleptic complex **1** was synthesized and characterized using X-ray diffraction crystallography, HRMS, and NMR spectroscopy (see supporting information). The complex** 2** was prepared following the procedure reported by Das et al.^15^

The X-ray diffraction structure shows that in **1**, the Ru^II^ center is closely held by two bipyridines and one phenanthroline unit with an average Ru–N distance of 2.07 Å (Figure S1, Table S1). It also shows, that although the carboxylic acid attached to the phenanthroline ligand is deprotonated to balance the overall charge in **1**, it does not coordinate with the metal center. Neither of the oxygens of the carboxylate unit are closer than 3.9 Å from the metal center excluding any possibility of direct Ru–O bond formation.^16^ In the case of **2,** **2 2**the average Ru–N (five pyridyl type units) distance is 2.03 Å and one of the oxygens of the carboxylate unit shows direct bonding interaction with the central ruthenium unit and with a Ru–O distance of 2.13(1) Å.^16^

The NMR spectroscopic details (Figure S2-6) and the mass envelopes in the HRMS experiments at 861.3401 (for **1**) and at 726.2331 (for **2**) further confirm (Figure S7 and S16) the molecular identity for **1** and **2** respectively. To study the formation of high valent ruthenium oxo species, acetonitrile was used as the major solvent, and ceric ammonium nitrate (CAN) {(NH₄)₂[Ce(NO₃)₆]} was used as a chemical oxidant. Either 10 or 20 equiv. of CAN (5 mM) were added at room temperature to an acetonitrile solution of **1** or **2** (0.5 mM) in an EPR tube, followed by rapid freezing in liquid nitrogen before measuring the EPR spectra at 10 K.

The need for excess CAN, a one-electron oxidant, for chemically induced water oxidation is reported by us and others.^11,12,17,18^

The EPR spectrum of **1** oxidized by CAN shows an almost axial signal (Fig. 2, **1**^**ox**^). The signal could be simulated with a single species with *g*_*x*_ = 2.72, *g*_*y*_ = 2.59, *g*_*z*_ = 1.43 (see Table S2 for more details). The signal is attributed to a Ru^III^ species with all N-donors in the coordination environment, similar to [Ru^III^(bpy)_3_]^3+^.^19^

**Fig. 2:**
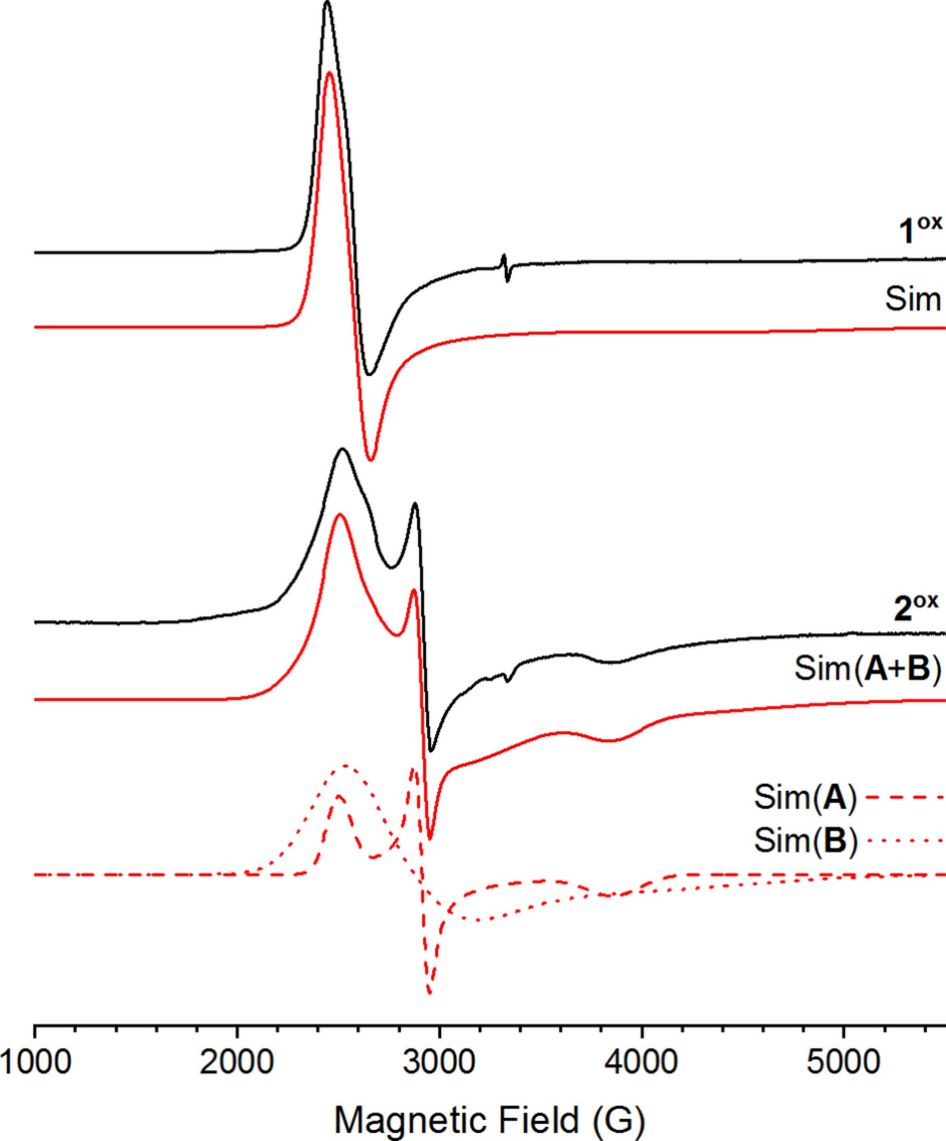
EPR spectra of **1** (0.5 mM) oxidized by 10 equiv. CAN (**1**^**ox**^) and **2** (0.5 mM) oxidized by 20 equiv. CAN (**2**^**ox**^) together with simulated spectra (red). Temperature: 10 K, microwave power: 200 µW (1^**ox**^) or 2 mW (2^**ox**^**).**

In contrast, the EPR spectrum of** 2** oxidized by CAN is more complex and shows signals from at least two different species. From the spectrum when 20 equiv. CAN was added (Fig. 2, **2**^**ox**^) the signal could be simulated as a combination of two species with rhombic EPR signals, species A with *g*_*x*_ = 2.66, *g*_*y*_ = 2.30, *g*_*z*_ = 1.73 and species B with *g*_*x*_ = 2.61, *g*_*y*_ = 2.27, *g*_*z*_ = 1.60 (see Table S2 for more details). From the simulation signal A corresponds to 24% of the total EPR spectrum, and signal B to 76%. The total intensity of the EPR signal of **2**^ox^ (Signal A + B) is comparable (ca 80%) to the intensity of the EPR signal from **1**^ox^ indicating that the species giving rise to signal A and B make up a significant part of **2** after addition of 20 eq CAN.

When 60 µl water was added to the EPR samples after the oxidation with CAN, for **2**, this led to a drastic reduction in the intensity of the EPR signals, especially of species A (Figure S9). This indicates that A is a high-valent Ru species that can react with water, instantaneously. In contrast, for **1** there was no change to the EPR spectrum upon the addition of an equal volume of water (Figure S8).

There are several reports of EPR-signals from Ru^V^ species in the literature (Table 1).^20–24^ While most of the reported Ru(V) signals are narrow with all g-values between 2.1 and 1.85, the EPR signal of [Ru^V^(O)(Py_2_^Me^tacn)]^3+^, (Py_2_^Me^tacn = *N*-methyl-*N,N*-bis(2-picolyl)-1,4,7-triazacyclononane), is similar to the signal from A. This species was electrochemically produced and characterized with in situ XAS confirming the high-valent state.^20^

**Table 1:** *g*-values associated with the EPR signals from **1** and **2** oxidized with CAN and selected Ru^III^ and Ru^V^ species from literature.

Ru-species	*g*_x_	*g*_y_	*g*_z_	ref
**1**^ox^	2.72	2.59	1.45	This work
A	2.66	2.30	1.73	This work
B	2.61	2.27	1.60	This work
[Ru^III^(bpy)_3_]^3+^	2.64	2.64	1.14	^19 ^
[Ru^III^(OH)(Py_2_^Me^tacn)]^3 +^ major	2.39	2.17	1.89	^20 ^
[Ru^III^(OH)(Py_2_^Me^tacn)]^3+^ minor	2.58	2.42	1.76	^20^
[Ru^V^(O)(Py_2_^Me^tacn)]^3+^	2.67	2.37	na	^20^
[Ru^V^(O)(O_2_COCEt_2_)_2_]^-^	2.08	1.98	1.91	^21^
[Ru^V^(O)(OH)(bpy)_2]_^2+^	2.07	2.00	1.87	^22^
[Ru^V^(O)(bda)(isoq)_2_]^+^	2.07	2.00	na	^23^
[Ru^V^(O)(pic)_2_(dpp)]^3+^	2.08	2.01	1.90	^24^

We propose that A is a Ru^V^-(Oxo) species that forms when the carboxylate unit on phenCO_2_ is protonated and decoordinated in the presence of acidic CAN (see further below), opening a coordination site for an oxo ligand, produced from trace water present in the acetonitrile. This results in an N_5_O coordination environment in **2** (Figure S13) which resembles the coordination environment in [Ru^V^(O)(Py_2_^Me^tacn)]^3+^ where the Py_2_^Me^tacn ligand coordinates the Ru with five N-donors and the oxo group is the sixth ligand.^25^ The second species, B, is a lot broader than A and also not as sensitive to the presence of water (Figure S9) and can be attributed to a Ru^III^ species with a hydroxo or hydroperoxo ligand.

DFT calculations [B97D3/6–31 + G(d)/SDD level] were used to understand the structural/electronic impact of the coordination sphere around the ruthenium center on the possible structure of Ru(V)-Oxo species. As a starting point, the structures of **1** and **2** were optimized and shown to reproduce essential features of the crystal structures (Table 2). In **2**, the oxygen atom of the carboxylate group binds to the ruthenium atom, resulting in the formation of a six-coordinated complex. This coordination enhances the overall stability and structural integrity of the complex in the Ru(II) state. Conversely, in **1**, the oxygen atom of the carboxylate group remains unbound to the ruthenium atom due to the saturated coordination environment created by the nitrogen atoms of the two ^tbu^bpy units and phenCO_2_ group, replicating the situation in the crystal structure.

**Table 2:** Relevant Ru–N and Ru–O bond lengths (DFT optimized).

Bond	Bond length (Å)		Bond	Bond length (Å)	
	**1**	**1** (Ru-Oxo)		**2**	**2** (Ru-Oxo)
Ru1-N1	2.06	2.18	Ru1-N1	1.98	1.99
Ru1-N2	2.06	2.12	Ru1-N2	2.07	2.05
Ru1-N3	2.06	2.07	Ru1-N3	2.07	2.10
Ru1-N4	2.11	**2.28**	Ru1-N4	1.98	2.17
Ru1-N5	2.06	**2.35**	Ru1-N5	2.07	2.18
Ru1-N6	2.07	2.13	Ru1-O1	2.14^a^	-
Ru1-O3	–	1.75^b^	Ru1-O3	–	1.72^b^

^a^Ru-carboxylate bond

^b^Ru = O bond.

The geometry of a Ru(V)-Oxo species generated from **2** could be optimized. This species has an additional oxo ligand (O3 in Fig. 3) coordinated to the Ru centre while the carboxylate group of phenCO_2_ is protonated and decoordinated. The bond length for Ru(V)-Oxo bond is calculated to be 1.72 Å. The average calculated Ru–N bond is ~ 2.10 Å with the Ru–N bonds in phenCO_2_ most elongated. It was not possible to optimize the structure of a Ru(V) species with **2** having acceptable Ru–N and Ru–O bond distances without including an oxo unit. We also conducted additional DFT calculations to investigate the energetics of the Ru^IV^(Oxyl) and Ru^IV^-O-Ce^III^(OH) species (Figures S18 and S19). However, despite multiple attempts, both calculations failed to converge, suggesting that these structures may be inherently unstable with **2**. Consequently, we expect both the I2M pathway (due to steric factors) and the I2M-HC pathway to be less favorable compared to the WNA mechanism.^4,8,9,26^

**Fig. 3:**
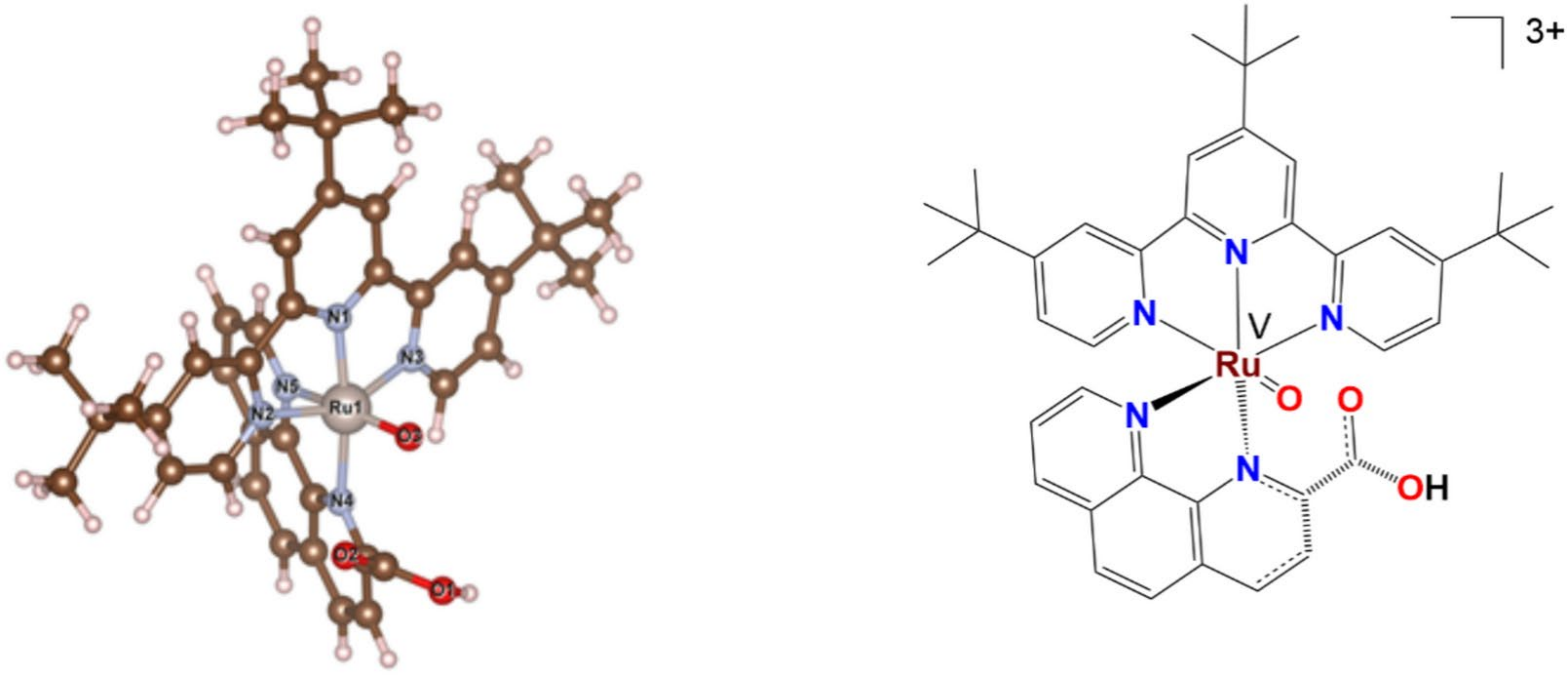
DFT [B97D3/6–31 + G(d)/SDD level] optimized structure of Ru(V)-Oxo species from **2** (left) and a pictorial representation of the same species (B) (right).

We also attempted to optimize a Ru(V) oxo species for **1** by creating a 7-coordinate species with an additional oxo ligand. An energy minimum was found with a Ru(V)-Oxo bond of 1.75 Å (Figure S17 and Table 2). In this species, the Ru–N bonds are elongated with a Ru–N average of ~ 2.18 Å. The phenCO_2_ ligand is almost decoordinated with Ru–N distances of 2.28 Å and 2.35 Å. This suggests that a hypothetical [(^tbu^bpy)_2_Ru^V^ (oxo) (phenCO_2_)]^2+^ species would be at the verge of decomposition and is in accordance with the lack of observations of high-valent species from **1** in both EPR and HRMS (see below). Low-temperature NMR spectroscopy (LTNMR) and rapid positive mode HRMS detection were used to investigate the existence of other relevant high-valent species. In the case of **1**, LTNMR (Figure S10, S11) in CD_3_CN} clearly shows the presence of the non-coordinated -COOH unit (at around 8.15 ppm) which gets sharper upon cooling it down from 20 to − 20 °C.

This species must have been generated through interaction with trace water present in in CD_3_CN. No such changes were observed in the case of **2** (Figure S14, S15). It is well known that NMR sensitivity increases with decreasing the temperature as the molecular motion in solution decreases.^27,28^ In this case, the proton exchange phenomenon of the carboxylic acid -OH of CD_3_CN dissolved **1** decreases with lowering the temperature to − 20 °C resulting in sharper signals.

The addition of 3.5 equiv. of CAN to complex **1** at –20 °C did not result in any significant changes, nor any new peaks were observed (Figure S11). However, in the case of complex **2**, the addition of an identical equivalent of CAN resulted in the immediate appearance of a sharp singlet at 11.9 ppm and a broader singlet at 3.2 ppm (Fig. 4, S15). The sharp singlet remains stable even at 20 °C, but upfield shifted by 0.8 ppm when compared to the signal at –20 °C. The singlet at 11.9 ppm and the other diamagnetic features of the spectrum point towards either an NMR active Ru^IV^-OH/Ru^IV^-OOH or Ru^II^-OH/Ru^II^-OOH species. Among these, Ru^IV^ species is more likely since such a downfield shift of the -OH/-OOH proton is unlikely with a Ru^II^ species.^25^

**Fig. 4:**
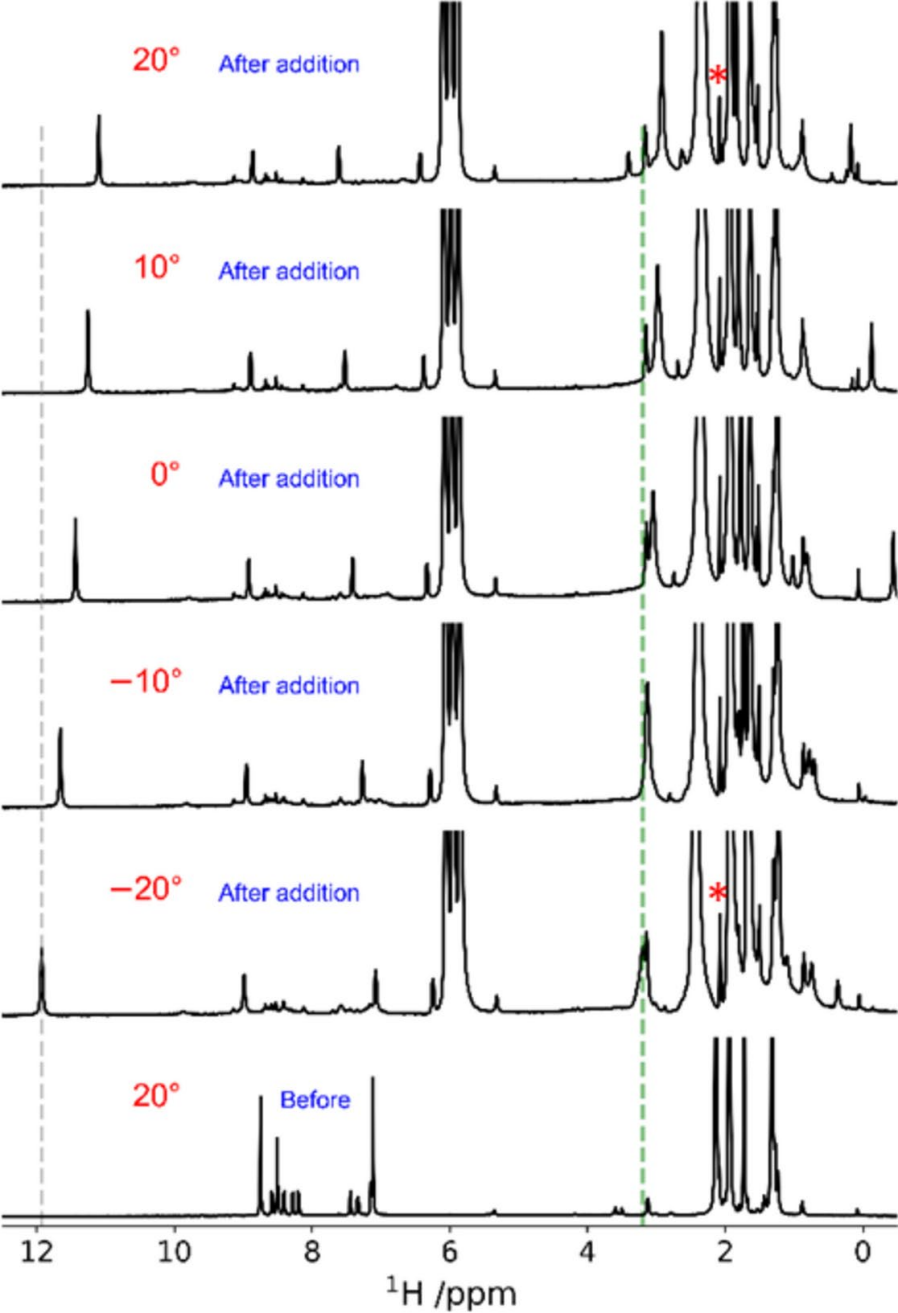
^1^H NMR spectra of **2** recorded in CD_3_CN at 20 °C before the addition, and at -20, -10, 0, 10, and 20 °C after the addition of 3.5 equiv. of CAN (The addition was performed at -20 °C, and spectra were recorded while the temperature was allowed to rise to 20 °C). * Represents the signal of water.

Another important observation is the unchanged intensity of the water signal at 2.1 ppm (Fig. 4, highlighted as *) between –20 and 20 °C after the addition of CAN, confirming that the Ru^IV^-OH/Ru^IV^-OOH species cannot participate in water oxidation. A further oxidized species, i.e., either Ru^V^ = O or Ru^IV^ O^·^ (unstable under these NMR conditions) is needed to oxidize water. The broader singlet at 3.2 ppm (at –20 °C) can be attributed to the partial opening and protonation of the carboxylate unit close to the metal center in the presence of acidic CAN. This signal is shifted upfield by 0.28 ppm at 20 °C compared to the spectrum at –20 °C. No new distinct peaks in the NMR signals were observed for **1** under identical reaction conditions that could be attributed to high-valent species (Fig. 4 and S11). Upon warming from −20 to 20 °C, for **1** in the presence of 3.5 equivalents of CAN, the only noticeable change was the conversion of a doublet at 8.2 ppm into a singlet at 8.1 ppm.

The rapid positive mode HRMS detection of the resulting solutions after CAN treatment of **2** shows two prominent peaks envelopes at 742.2274 and 759.1953 which are attributed to [(^tbu^tpy)Ru^IV^(= O)(phenCO_2_)]^+^ and[(^tbu^tpy)Ru^III^(OOH)(phenCO_2_)]^+^ respectively (Figure S16). This also points towards a species related to Ru^IV^-OH/ Ru^IV^-OOH that was observed in the LTNMR spectroscopy (see above). No such corresponding high valent Ru^IV^ species could be found after the CAN treatment on **1** (Figure S12), only a single electron oxidized Ru^III^ species is observed as a new peak envelope at 431.1731.

The UV–Vis spectroscopic investigation was carried out at room-temperature (23 °C) using 2–15 equivalents of CAN in the presence of 0.15 mM of **1** and **2** in dry MeCN (Fig. 5). For **1**, with 2 equivalents of CAN, significant spectral changes were observed, including the disappearance of the MLCT and π–π* bands at approximately 455 nm and 250 nm, respectively, and the appearance of multiple peaks in the 260–350 nm range. These observations correlate with possible metal leaching and bipyridine ligand leaching under the oxidative environment. In the case of **2**, a transition to a new species was observed upon the addition of CAN. The species is characterized by an MLCT band at 465 nm and a new π–π* transition band at 325 nm and has maximum intensity after the addition of 8 equivalents of CAN. This is likely attributable to the formation of a high-valent ruthenium-oxo species, tentatively identified as Ru(V)/Ru(VI), similar to the findings reported by Kundu et al.^26^ However, the intensity of this new absorption maximum diminished with the further addition of CAN up to a total of 15 equivalents, suggesting its consumption due to oxidation of trace water in the reaction mixture or slow decomposition under excess CAN at room temperature.

**Fig. 5:**
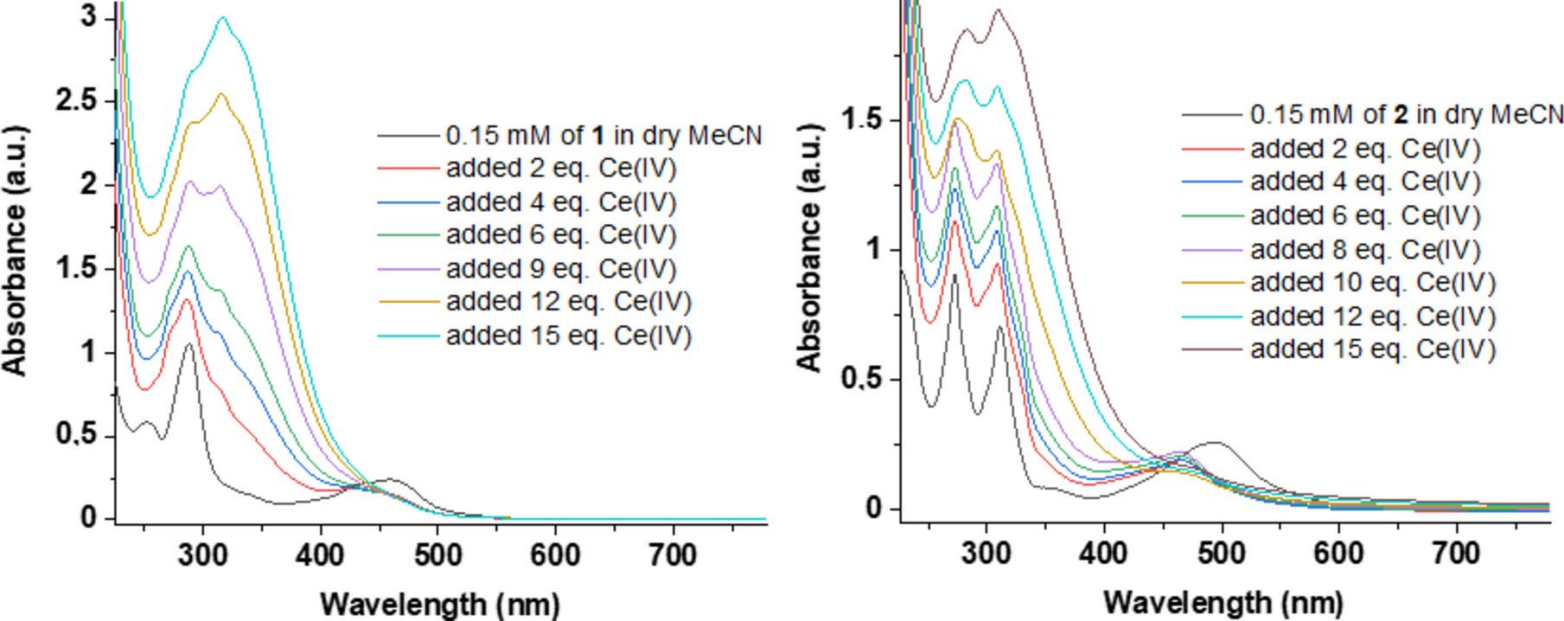
UV–Vis spectroscopic investigation at room temperature (23 °C) using 2–15 equivalents of CAN in the presence of 0.15 mM of **1** (**A**) and **2** (**B**) in dry MeCN.

In summary, we report different behaviour of two quite identical heteroleptic ruthenium complexes (**1** and **2**) under oxidative conditions. Both complexes are coordinatively saturated, each having six coordination sites and a distorted octahedral geometry around the Ru centres. In the case of **1**, the six coordination sites on ruthenium filled by two bipyridines and one phenanthroline where the -COOH group on phenanthroline-carboxylic acid remains non-coordinated. Whereas, in case of **2**, the ruthenium is coordinated by a terpyridine, and a phenanthroline carboxylate. In this case, the carboxylate unit is directly bonded to the central ruthenium as the sixth donor.

In the case of **1**, it was impossible to accommodate an incoming water molecule, as bipyridine does not exhibit any hemilabile behaviour. However, in **2**, the carboxylate coordination from the phenCO₂ unit allows for such interaction due to its proton-and electron-dependent (PCET type) hemilabile nature. These structural features in **2** allowed us to generate and characterize several high-valent Ru species; a Ce^IV^-induced Ru(V)-Oxo intermediate (by EPR and supported by UV–Vis spectroscopy), Ru^IV^-hydroxo/peroxo species (by LTNMR), and Ru^IV^–oxo + Ru^III^-peroxo species (by HRMS). The assignment of the Ru(V)-Oxo intermediate is primarily based on EPR findings, supported by additional spectroscopic data, previously reported systems, and our unsuccessful attempts to optimize a Ru(V) species without the oxo unit.

No similar species could be generated or detected with **1** due to the unavailability of any vacant coordination site on the ruthenium center. Only a single electron-oxidized Ru^III^ species is observed (by EPR and HRMS). This demonstrates the substantial difference between having and not having the possibility to bind the flexible carboxylate unit to the ruthenium center. To the best of our knowledge, this comparison of two very closely related systems is being discussed for the first time. Another important finding from the combined LTNMR and EPR spectra is that with these ligand frameworks, the Ru^IV^-OH/ Ru^IV^-OOH species (from **2**) cannot participate in water oxidation and presumably it has to be further oxidized i.e., either Ru^V^ = O or Ru^IV^-O^·^ to actively participate in water oxidation. DFT calculations propose a potential structure for a Ru(V)-Oxo species generated from **2** but suggest that high-valent Ru(V)-Oxo species from **1** would exhibit highly elongated Ru–N bond distances, potentially leading to the decomposition of the complex under oxidative conditions.

## Supplementary Information

Supplementary Information.

## Data Availability

Data is provided within the manuscript or supplementary information files.
