# Supplementary material for: Carboxylate and coordination influence on the formation of an active RuV Oxo species
Source: Sci Rep. 2025 Feb 18;15:5882. doi: 10.1038/s41598-025-89062-5 (PMC11836329; doi:10.1038/s41598-025-89062-5)
Supplement: Supplementary file 1 — Supplementary Information. [file 41598_2025_89062_MOESM1_ESM.docx]

Supporting information for

Carboxylate and Coordination Influence on the Formation of an Active Ru(V) Oxo species

Jamal El-Abid,^†^ Kevin M. Dorst,^†^ A. Ken Inge,^ŧ^ Oscar Verho,^γ^ Varun Kundi,^Ƭ^ Priyank V. Kumar,^Ƭ^ Anders Thapper,^‡*^ Biswanath Das^†*^

†Department of Organic Chemistry, Arrhenius Laboratory Stockholm University, Svante Arrhenius väg 16C, 10691 Stockholm, Sweden

ŧ Department of Materials and Environmental Chemistry, Stockholm University, Svante Arrhenius väg 16C, 106 91 Stockholm, Sweden

^γ^Department of Medicinal Chemistry, Biomedicinskt Centrum BMC, Uppsala University, SE-75123 Uppsala, Sweden

^Ƭ^School of Chemical Engineering, The University of New South Wales, Sydney, NSW 2052, Australia

^‡^Department of Chemistry – Ångström Laboratory, Uppsala University, P.O. Box 523, SE-75120, Uppsala, Sweden

Corresponding authors – [biswanath.das@su.se](mailto:biswanath.das@su.se) and anders.thapper@kemi.uu.se

**1. General considerations**

**Materials and Methods:**

Unless otherwise noted, all reagents and solvents were commercially available and used as received from Thermo Fischer, TCI, and Sigma Aldrich. Reagent grade organic solvents were used for the purification and HPLC grade solvents were used for synthesis and other operations.

**NMR spectra** were recorded on a Bruker Avance II 500 MHz spectrometer using a 5 mm BBO probe equipped with Z-gradients. Peaks were referenced to the internal solvent peaks (δH 3.31 for MeOD).

**HRMS spectra** were recorded in positive mode on a Bruker Daltonics MicrOTOF mass spectrometer using electrospray ionization as the ion source. The peaks were referenced to a serially injected sample of 10 mM sodium formate. The data were exported and presented using GNU Octave. ESI-HRMS measurements were performed using the Bruker Daltonics microTOF mass spectrometer (direct injection, positive mode, all samples in MeOH).

**EPR Spectroscopy** measurements were performed at 10 K using a Bruker ESR-500 spectrometer equipped with an ER 4122SHQ resonator, an ESR900 cryostat, and an Oxford ITC503 temperature controller. EPR parameters: Microwave frequency, 9.38 GHz; modulation frequency, 100 kHz. EPR samples were prepared at room temperature by adding 10 or 20 equivalents of CAN (30 or 60 µl, final conc. 5 and 10 mM, respectively) dissolved in acetonitrile to an acetonitrile solution of **1** or **2** (150 µl, 0.5 mM) in an EPR tube, followed by rapid freezing in liquid nitrogen. Additional samples were prepared where water (60 µl, ~25 v/v%) were added to the oxidized samples afterwards or where 10 equivalents CAN (30 µl, final conc. 5 mM) dissolved in water was added to an acetonitrile solution of **1** or **2** (150 µl, 0.5 mM. EPR spectra were simulated using Easyspin 5.2.28.^1^

**X-ray diffraction analysis:**

Single crystal X-ray diffraction data on a suitable crystal of compound [(^tbu^bpy)_2_Ru^II^(phenCO_2_)](PF_6_)·H_2_O (**1**) were collected using Mo Kα radiation on a Bruker D8 VENTURE diffractometer equipped with a PHOTON III detector. The dataset was reduced and absorption correction was applied using the APEX3 suite. The crystal structures were solved and refined by SHELXT and SHELXL respectively.^2^ The crystal structures were refined using full-matrix least-squares based on F^2^ with all non-hydrogen atoms anisotropically defined. Hydrogen atoms on the complex were placed using a riding model. Hydrogen atoms on the water molecule were located in the difference map and restrained. Restraints were applied to anisotropic ADPs of disordered t-butyl groups and the PF_6_ anion. A summary of the crystallographic data and refinement parameters is provided in Table S1. Crystals of (**1**) contain solvent accessible voids with 116 electrons found and treated using the SQUEEZE procedure by PLATON.^3^ These are attributed to disordered solvent molecules of water/ethanol.

CCDC 2282842 contains the supplementary crystallographic data for this paper. These data can be obtained free of charge from The Cambridge Crystallographic Data Center via <http://www.ccdc.cam.ac.uk/structures>.

**2. Synthesis:**

(^tbu^bpy)_2_RuCl_2_ was prepared following a well-known procedure reported by Meyer et.al.^4^

[(^tbu^tpy)Ru^II^(phenCO_2_)](PF_6_) (**2**) was prepared following a reported procedure from Das et.al.^5^

**Synthesis of** [(^tbu^bpy)_2_Ru^II^(phenCO_2_)](PF_6_) (**1**)**:**

The ruthenium complex [(^tbu^bpy)_2_Ru^II^(phenCO_2_)](PF_6_) (**1**) was prepared by refluxing a 50 ml ethanol-water (4:1) solvent mixture having 0.25 g (0.353 mmol) of (^tbu^bpy)_2_RuCl_2_, 0.079 g (0.354 mmol) of 1,10-phenanthrolin-2-carboxylic acid (phenCO_2_) and 0.3 ml of triethyl amine ( 2.2 mmol). The solution was monitored by TLC and was refluxed for 6 h followed by the addition of 1 ml of concentrated aqueous KPF_6_ solution. Cooling down of the reaction mixture to room temperature resulted in a reddish-orange color precipitate of **1** (0.292 g, 82%). The precipitate was collected and washed three times with 5 ml of ice-cold water, followed by recrystallization by slow evaporation of an acetone solution, to get shining orange color crystals of **1**. See all the characterization in section 3 and 4.

**3. X-ray diffraction structure of** [(^tbu^bpy)_2_Ru^II^(phenCO_2_)](PF_6_)·H_2_O (**1**):


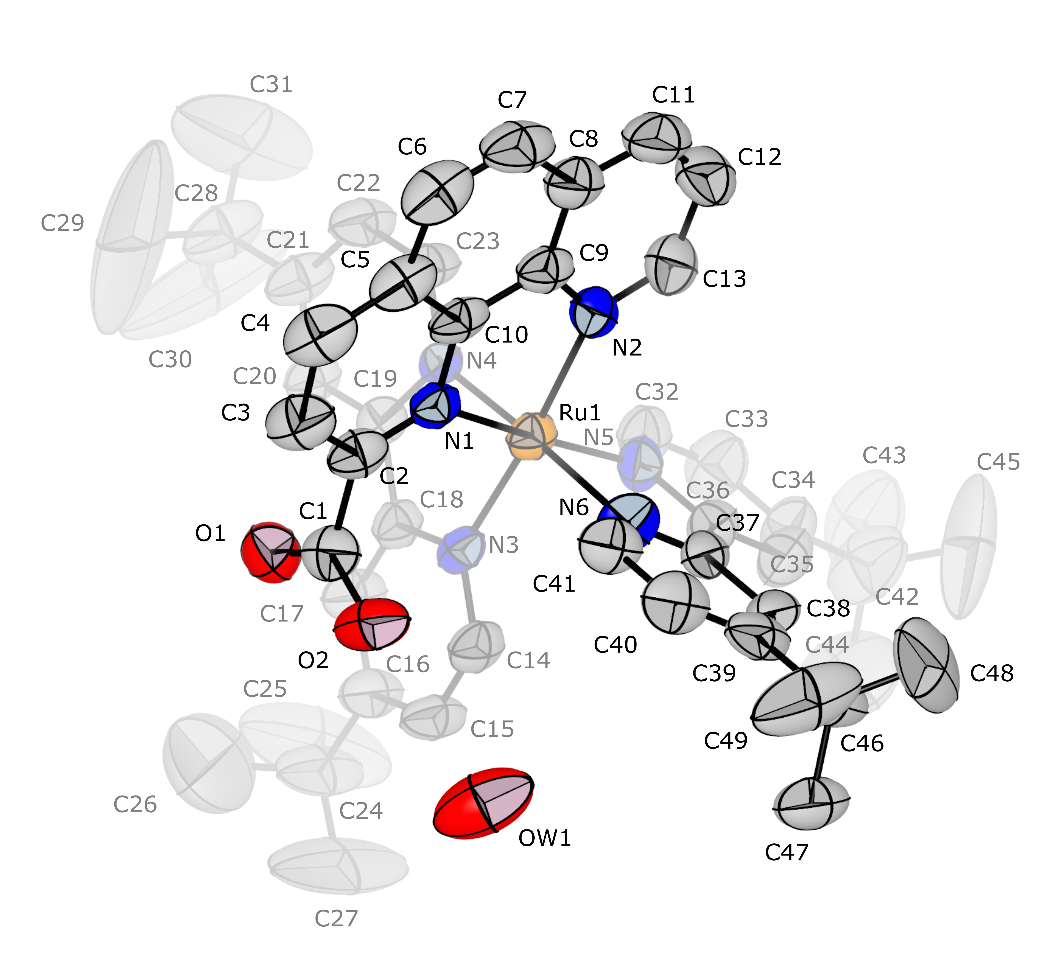


**Figure S1**. Structure of [(^tbu^bpy)_2_Ru^II^(phenCO_2_)](PF_6_)·H_2_O (CCDC 2282842) as determined by single crystal X-ray diffraction. Atoms are colored as follows: carbon (grey), oxygen (red), nitrogen (blue), ruthenium (orange), phosphorus (purple), and fluorine (green). PF_6_^-^ anion and hydrogen atoms are omitted for clarity. The ellipsoid contour is shown at the 50% probability level.

**Table S1:** Crystallographic data and refinement details for [(^tbu^bpy)_2_Ru^II^(phenCO_2_)](PF_6_)·H_2_O (**1**)

| CCDC deposition number | 2282842 |
| --- | --- |
| Empirical formula | C49 H57 F6 N6 O3 P Ru |
| formula weight | 1024.04 |
| temperature | 300 K |
| wavelength | 0.71073 Å |
| crystal system | monoclinic |
| space group | *P*2_1_/*c* (No. 14) |
| unit cell dimensions | *a* = 12.3494 (8) Å  *b* = 34.672 (2) Å  *c* = 12.5407 (9) Å  *β* = 91.366 (2) ° |
| volume | 5368.2 (6) Å^3^ |
| *Z* | 4 |
| density (calculated) | 1.267 g/cm^3^ |
| absorption coefficient | 0.385 mm^-1^ |
| *F*(000) | 2120 |
| *θ* range for data collection | 2.005 ° to 25.525° |
| index ranges | -13≤*h*≤14, -41≤*k*≤41, -15≤*l*≤15 |
| reflections collected | 99265 |
| independent reflections | 9932 [*R*(int) = 0.1223] |
| absorption correction | multi-scan |
| data / restraints / parameters | 9932 / 84 / 603 |
| goodness-of-fit on *F*^2^ | 1.156 |
| final R indices [*I*>2*σ*(*I*)] | *R*1 = 0.1174, w*R*2 = 0.2696 |
| largest diff. peak and hole | 1.343 and –0.967 e/Å^3^ |

**4. NMR, HRMS, and EPR characterization of 1 and 2:**

**Figure S2**. Chemical structure of complex [(^tbu^bpy)_2_Ru(phenCO_2_)](PF_6_) **(1)**

^1^H NMR (400 MHz, CD_3_CN) δ 8.48 (d, *J* = 8.2 Hz, 1H), 8.44 (d, *J* = 8.1 Hz, 1H), 8.40 (s, 1H), 8.39 (s, 1H), 8.36 (s, 1H), 8.28 (d, *J* = 6.1 Hz, 1H), 8.22 (s, 1H), 8.15 – 8.07 (m, 2H), 7.72 – 7.68 (m, 1H), 7.60 (d, *J* = 6.0 Hz, 1H), 7.57 (dd, *J* = 8.3, 5.4 Hz, 1H), 7.48 (d, *J* = 8.3 Hz, 1H), 7.36 (d, *J* = 6.1 Hz, 1H), 7.29 (m, 2H), 7.15 (dd, *J* = 6.1, 2.0 Hz, 1H), 7.05 (dd, *J* = 6.2, 2.0 Hz, 1H), 6.96 (d, *J* = 6.0 Hz, 1H), 1.37 (s, 12H), 1.34 (s, 16H), 1.32 (s, 8H).

^13^C NMR (101 MHz, CD_3_CN) δ 207.4, 163.1, 162.6, 162.5, 161.6, 159.4, 158.5, 158.2, 157.6, 154.9, 153.8, 152.4, 151.5, 150.9, 148.8, 137.9, 136.8, 131.7, 129.9, 129.1, 127.2, 125.9, 125.3, 124.5, 124.4, 124.3, 123.2, 122.0, 121.7, 121.1, 120.2, 30.8, 30.5, 30.4.


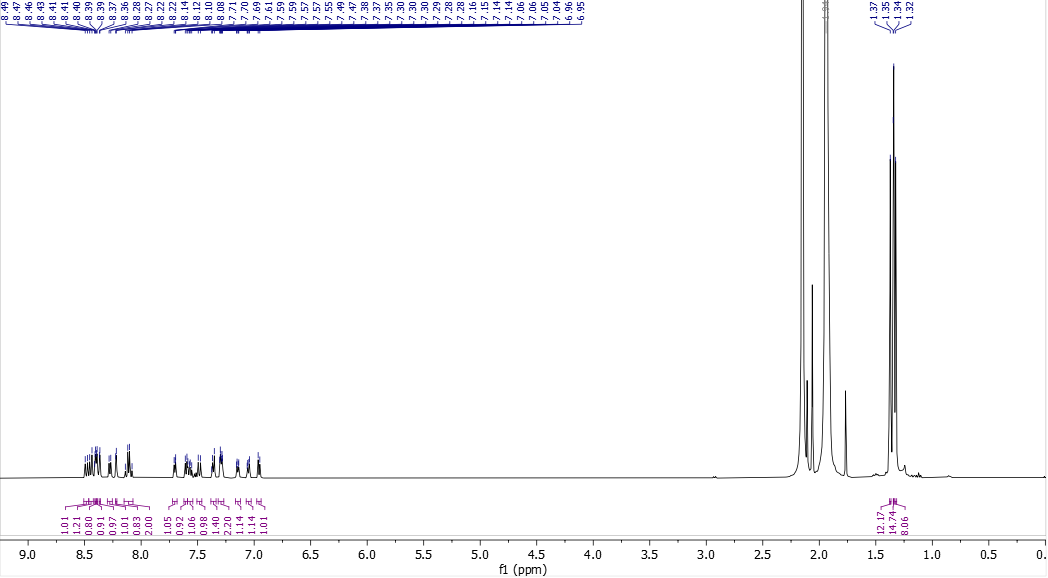


**Figure S3**. ^1^H NMR spectrum of **1** in CD_3_CN.


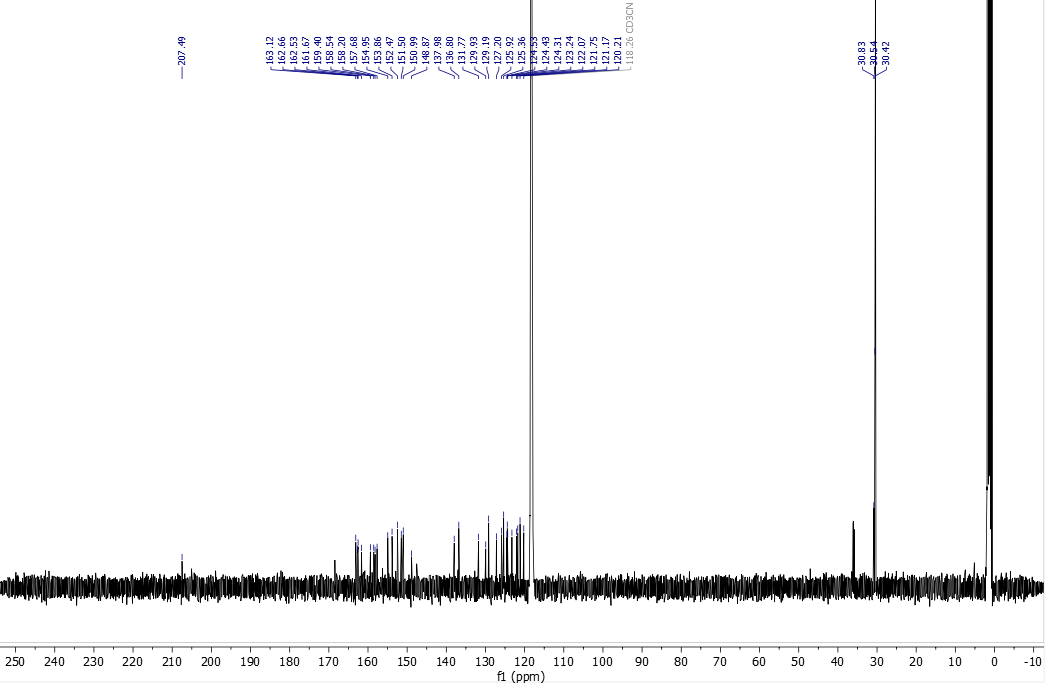


**Figure S4.** ^13^C NMR spectrum of **1** in CD_3_CN.


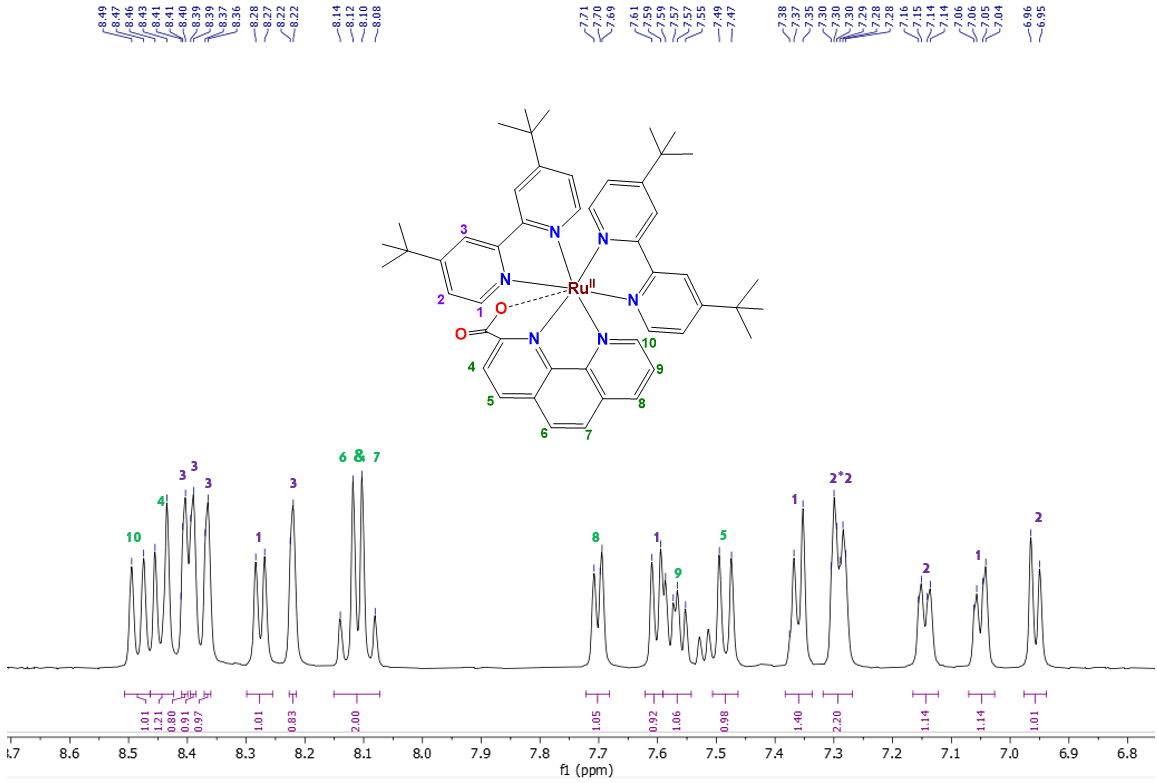


**Figure S5.** Assignment of ^1^H NMR spectrum of **1** in CD_3_CN.


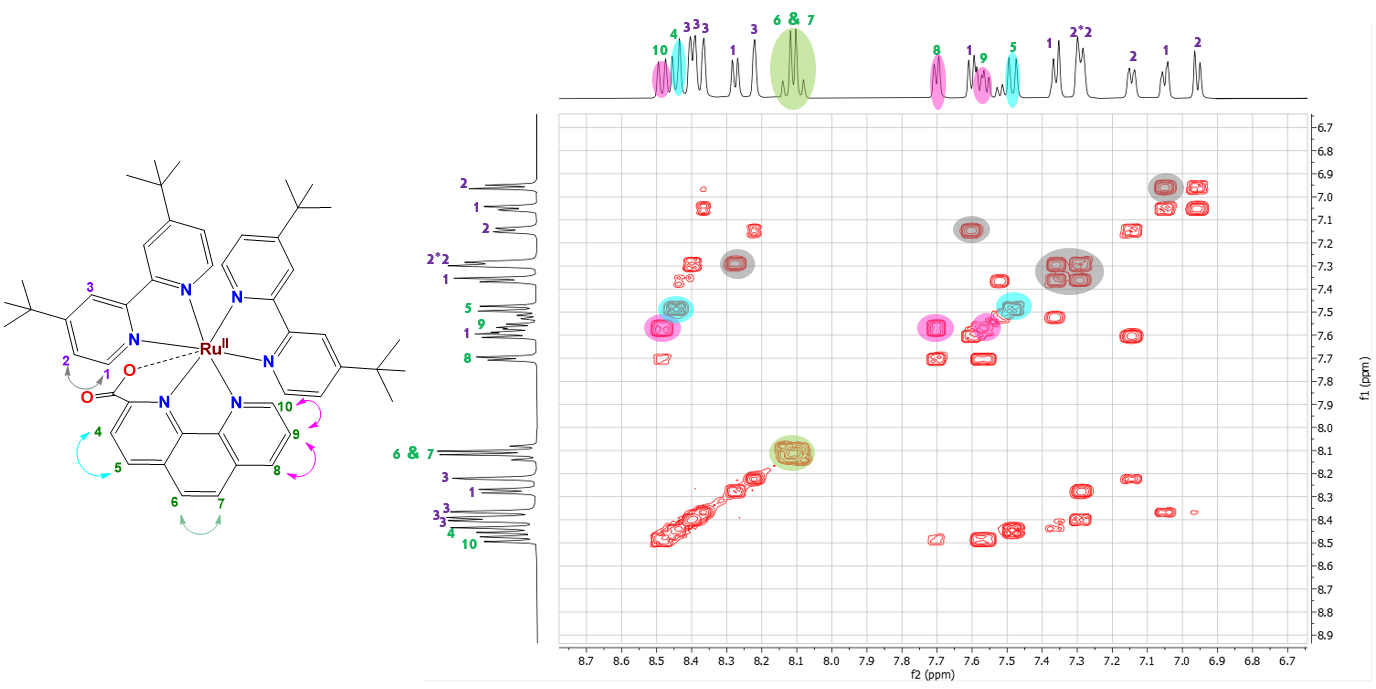


**Figure S6.** ^1^H-^1^H COSY spectrum of **1** in CD_3_CN.


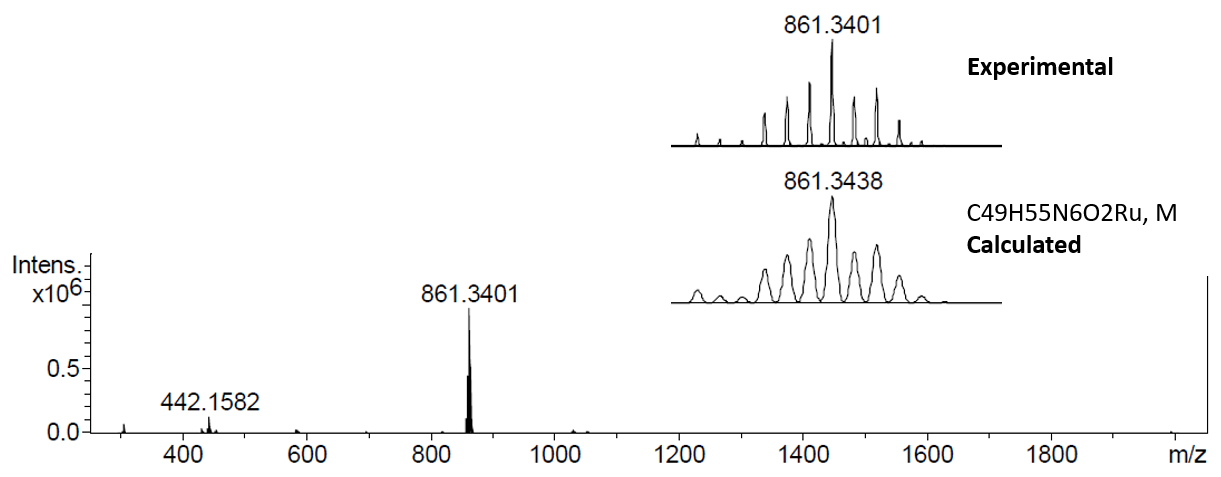


**Figure S7**. HRMS spectrum (Done in acetonitrile), and the experimental and calculated isotopic patterns of the **1**. HRMS [M]: calcd. For C_49_H_55_N_6_O_2_Ru, 861.3438; found 861.3401.


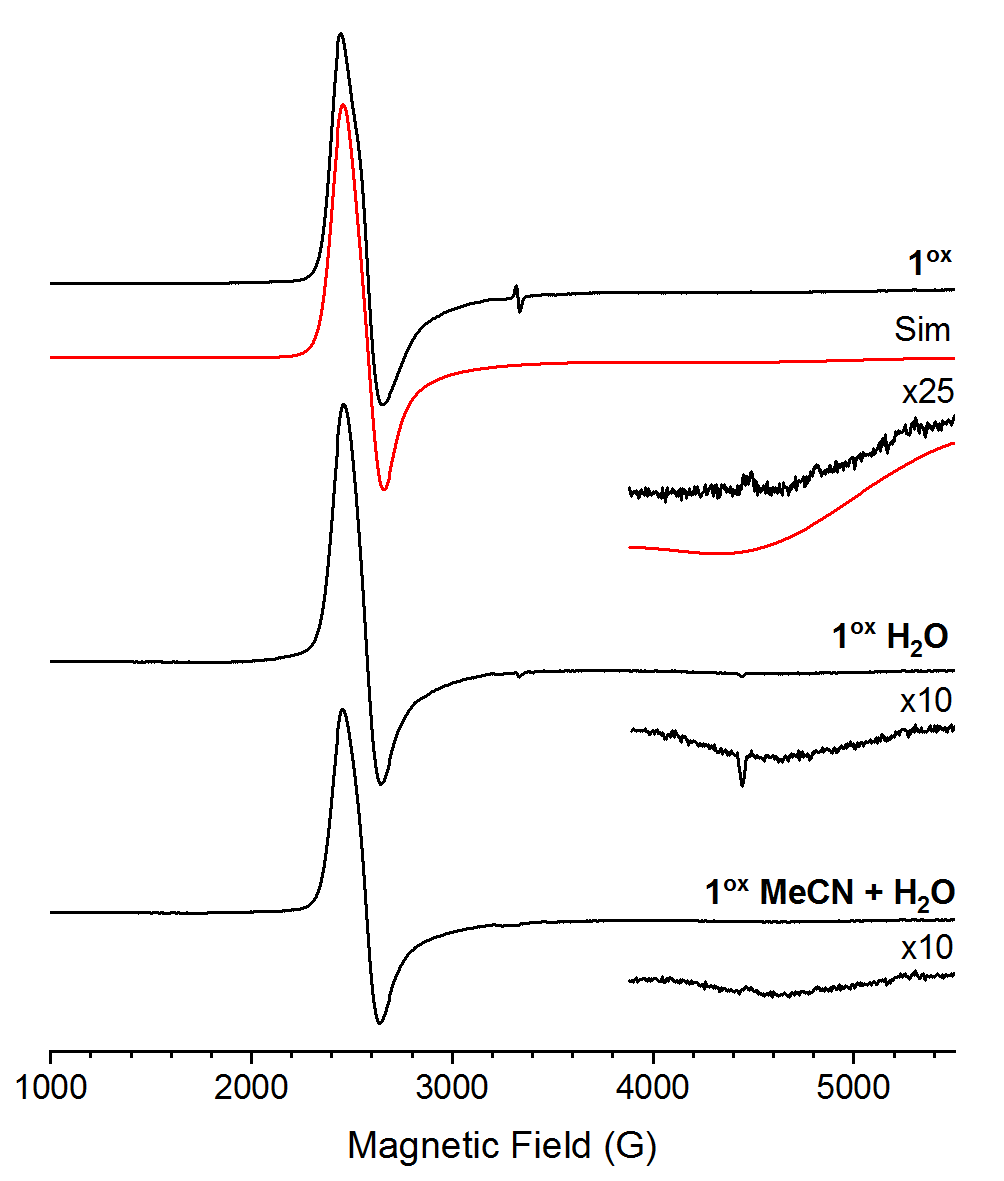


**Figure S8.** EPR spectra of **1** oxidized by 10 eq. CAN dissolved in MeCN (**1^ox^**), **1** oxidized by 10 eq. CAN dissolved in H_2_O (**1^ox^ H_2_O**), and **1** oxidized by 10 eq. CAN dissolved in MeCN followed by the addition of 60 µL (~25 v/v%) H_2_O (**1^ox^ MeCN + H_2_O**). [**1**] = 0.5 mM in acetonitrile. The red spectrum is a simulation of **1^ox^**, see table S2 for details. The insets show magnifications of the high-field region where *g_z_* is observed. Temperature: 10 K, microwave power: 200 µW.


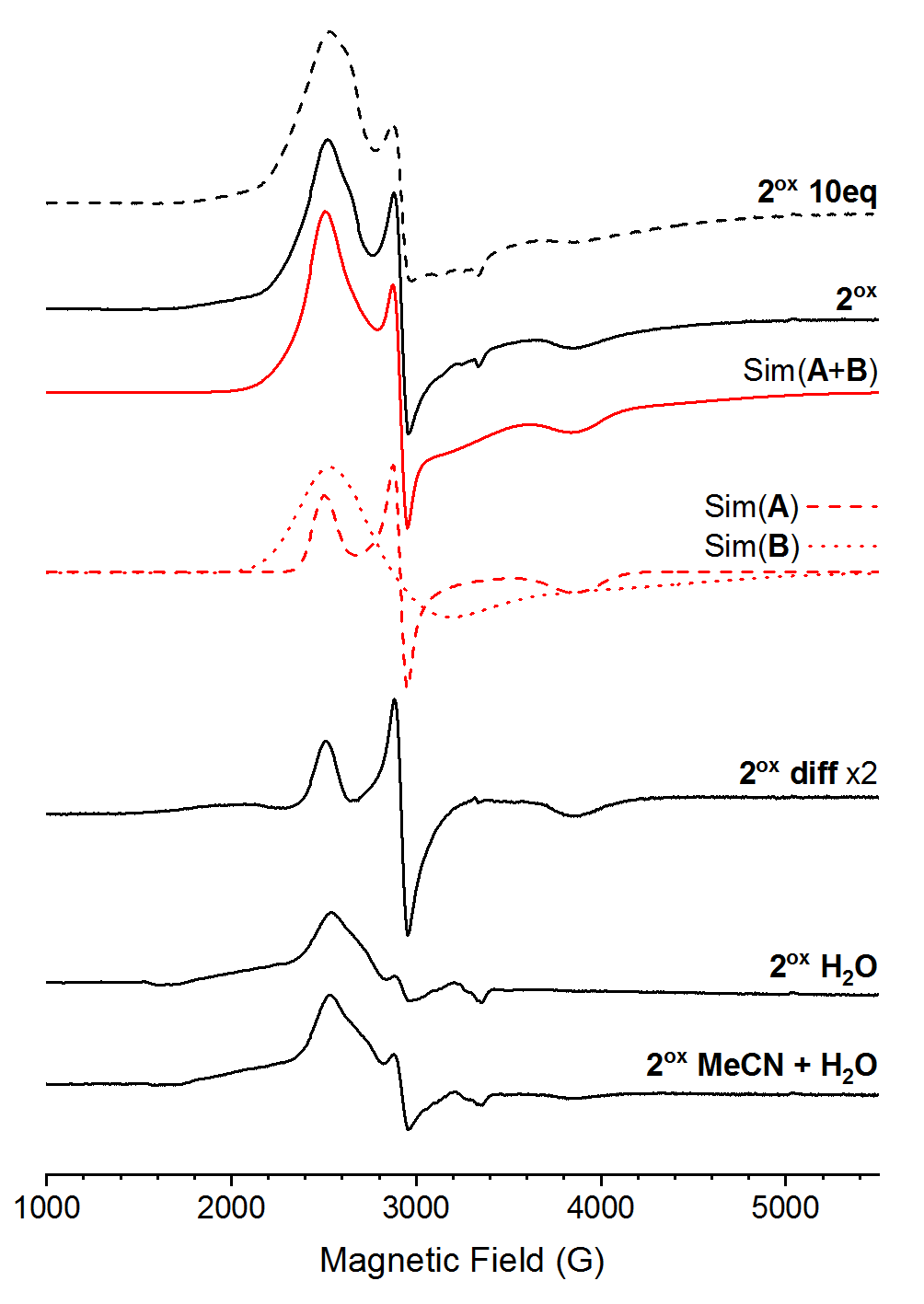


**Figure S9.** From top to bottom: EPR spectra of **2** oxidized by 10 eq. (**2^ox^ 10eq**) or 20 eq. (**2^ox^**) CAN dissolved in MeCN. Simulations of **2^ox^** (Sim **A+B**), **A**, and **B** (red lines), see table S2 for details.
A difference spectrum of (**2^ox^**) – 0.8*(**2^ox^ 10eq**) magnified 2 times to isolate species **B** (**2^ox^ diff**). EPR spectra of **2** oxidized by 10 eq. CAN dissolved in H_2_O (**2^ox^ H_2_O**), and **2** oxidized by 10 eq **CAN** dissolved in MeCN followed by the addition of 60 µL (~25 v/v%) H_2_O (**2^ox^ MeCN + H_2_O**). [**2**] = 0.5 mM in acetonitrile. Temperature: 10 K, microwave power: 2 mW.

**Table S2.** Parameters for the simulations of EPR signals from species **1^ox^**, **A**, and **B**. **A** and **B** were simulated together in a 2-component least square refinement of **2^ox^**. Hyperfine couplings were not resolved for any of the species and was simulated together with anisotropic line broadening using H-strain.

| Species | *g*-values | | | H-strain | | |
| --- | --- | --- | --- | --- | --- | --- |
|  | *g_x_* | *g_y_* | *g_z_* | H_x_ | H_y_ | H_z_ |
| **1^ox^** | 2.72 | 2.59 | 1.45*^a^* | 420 | 440 | 2500 |
| **A** | 2.66*^b^* | 2.30*^b^* | 1.73*^b^* | 540 | 215 | 750 |
| **B** | 2.61 | 2.27 | 1.60*^c^* | 1310 | 1760 | 3000*^c^* |

*a*; Determined from EPR-spectrum of **1^ox^** and fixed in least square refinement, *b*; values obtained from simulation of **2^ox^ diff** and fixed in the simulation of A+B, *c*; not well resolved and fixed in least square refinement.


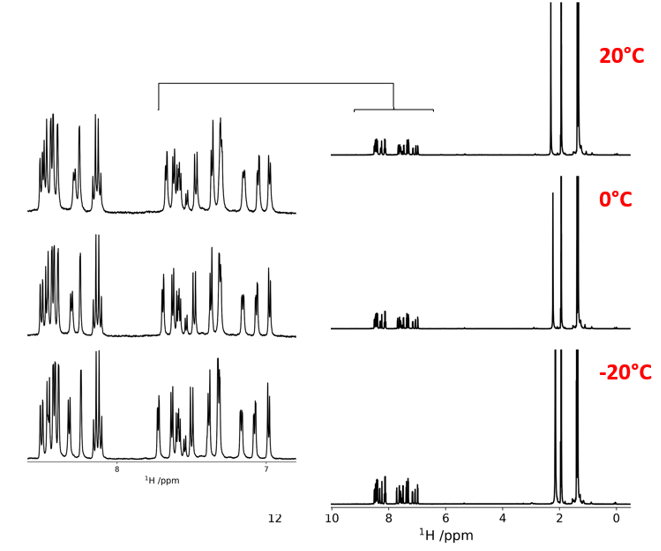


**Figure S10**. ^1^H NMR spectra of **1** recorded in CD_3_CN at -20, 0 and 20°C.


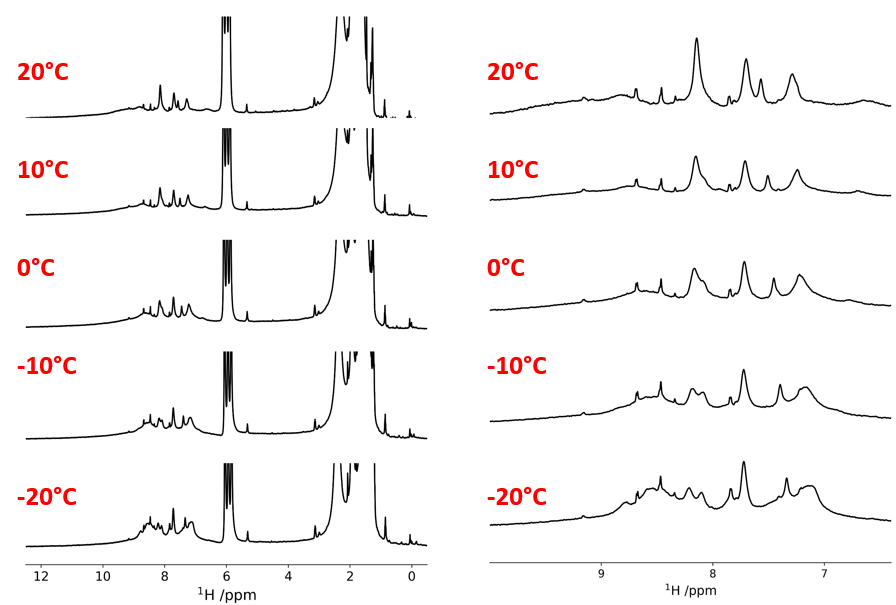


**Figure S11**. ^1^H NMR spectra of complex **1** recorded in CD_3_CN at -20, -10, 0, 10, and 20°C after the addition of 3.5 equivalent of (NH₄)₂[Ce(NO₃)₆] (The addition was performed at -20°C, and spectra were recorded while the temperature was allowed to rise up to 20°C).


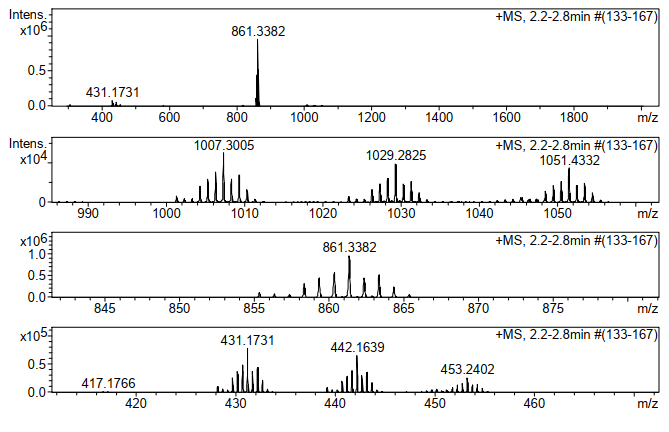


**Figure S12**. HRMS spectrum (Done in acetonitrile), after addition of 3.5 equivalent of (NH₄)₂[Ce(NO₃)₆] to **1**. No peaks corresponding to [(^tbu^bpy)_2_Ru (=O)(phenCO_2_)]^+^ or [(^tbu^bpy)_2_Ru(OOH)(phenCO_2_)]^+^ were visible.

**Complex 2**:

**Figure S13**. Chemical structure of complex [(^tbu^tpy)Ru(phenCO_2_)](PF_6_) (**2**)


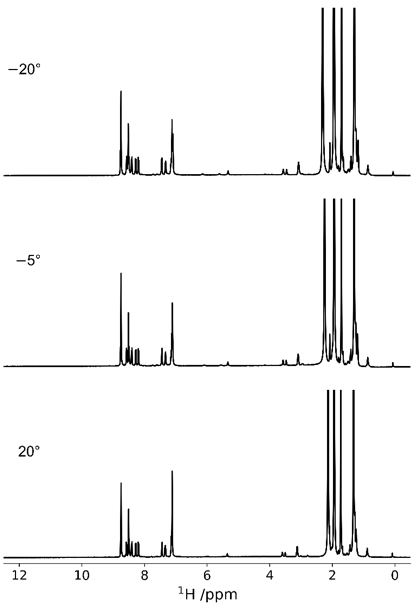


**Figure S14**. ^1^H NMR spectra of **2** recorded in CD_3_CN at -20, -5 and 20°C.


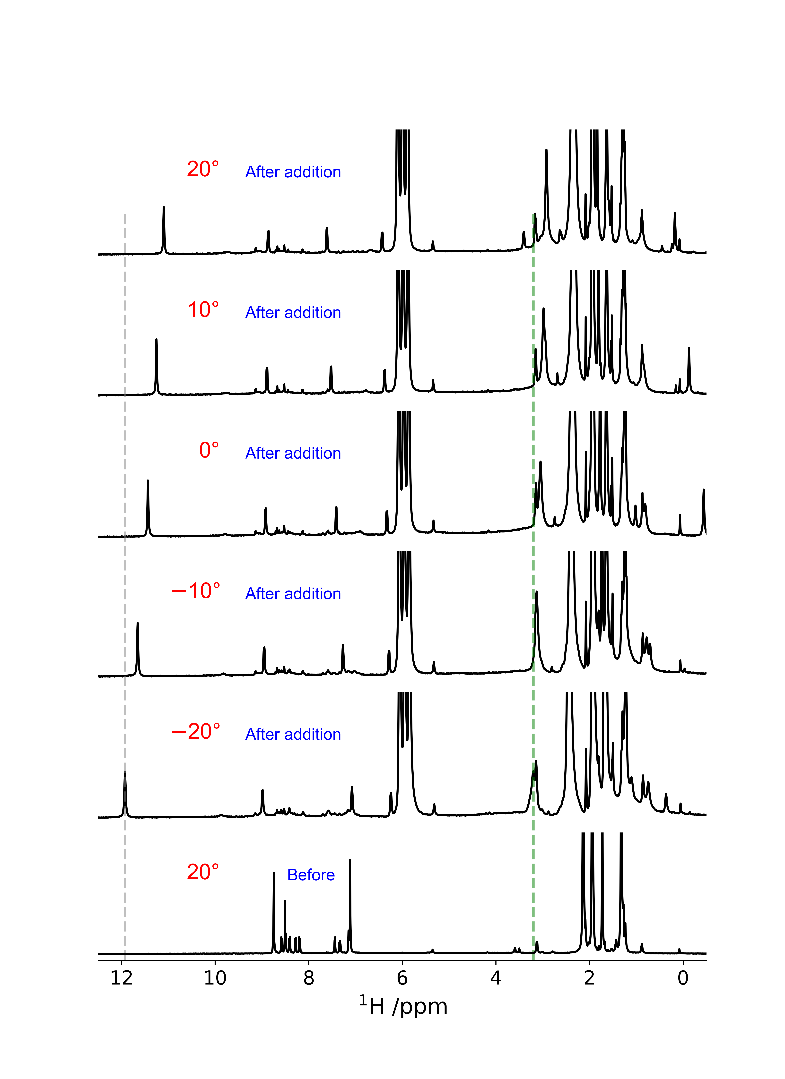


**Figure S15**. An enlarged version of the ^1^H NMR spectra of **2** recorded in CD_3_CN at 20°C before the addition, and at -20, -10, 0, 10 and 20°C after the addition of 3.5 equivalent of (NH₄)₂[Ce(NO₃)₆] (The addition was performed at -20°C, and spectra were recorded while the temperature was allowed to rise to 20°C).


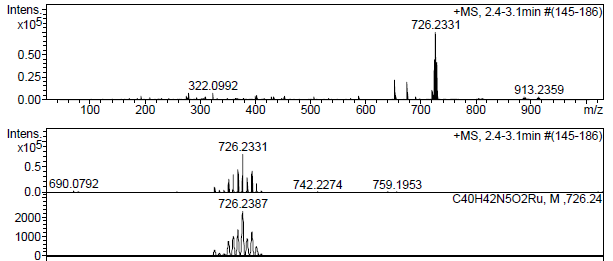


The experimental and calculated isotopic patterns observed of newly species.

- At m/z 742.2274 which correspond to **[(^Bu^tpy)Ru(=O)(phenCO_2_)]^+^**


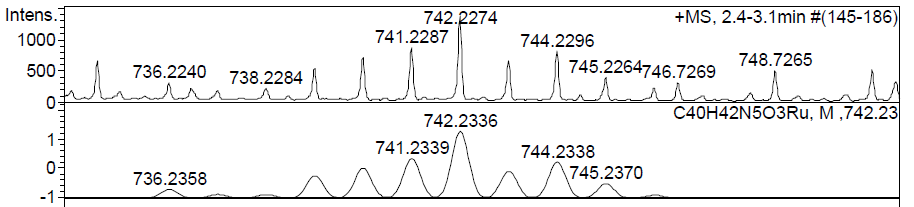


- At m/z 759.1953, which correspond to **[(^Bu^tpy)Ru(OOH)(phenCO_2_)]^+^**


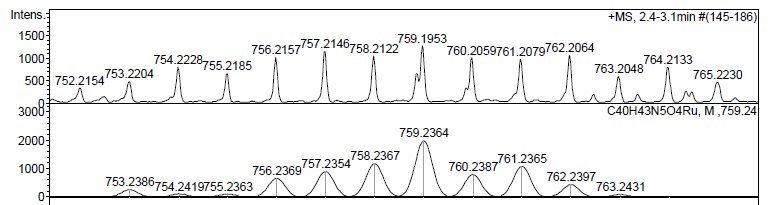


**Figure 16**. HRMS spectrum after the addition of 3.5 equivalent of (NH₄)₂[Ce(NO₃)₆] to the complex **2**.

**Computational details:**

All calculations were performed using Gaussian 16.^6^ Geometry optimizations were conducted at the B97D3/6-31+G(d)/SDD level of theory.^7^ The 6-31+G(d) basis set was employed for C, H, O, and N atoms, while the SDD basis set was used for the Ru atom. Vibrational frequency calculations were performed at the same level of theory to confirm optimized geometries as true minima with zero imaginary frequencies. The SMD implicit solvation model^8^ was utilized to account for the solvent effects of acetonitrile. Convergence criteria for both optimizations and frequency calculations were set to the default values provided by Gaussian 16.^6^

| 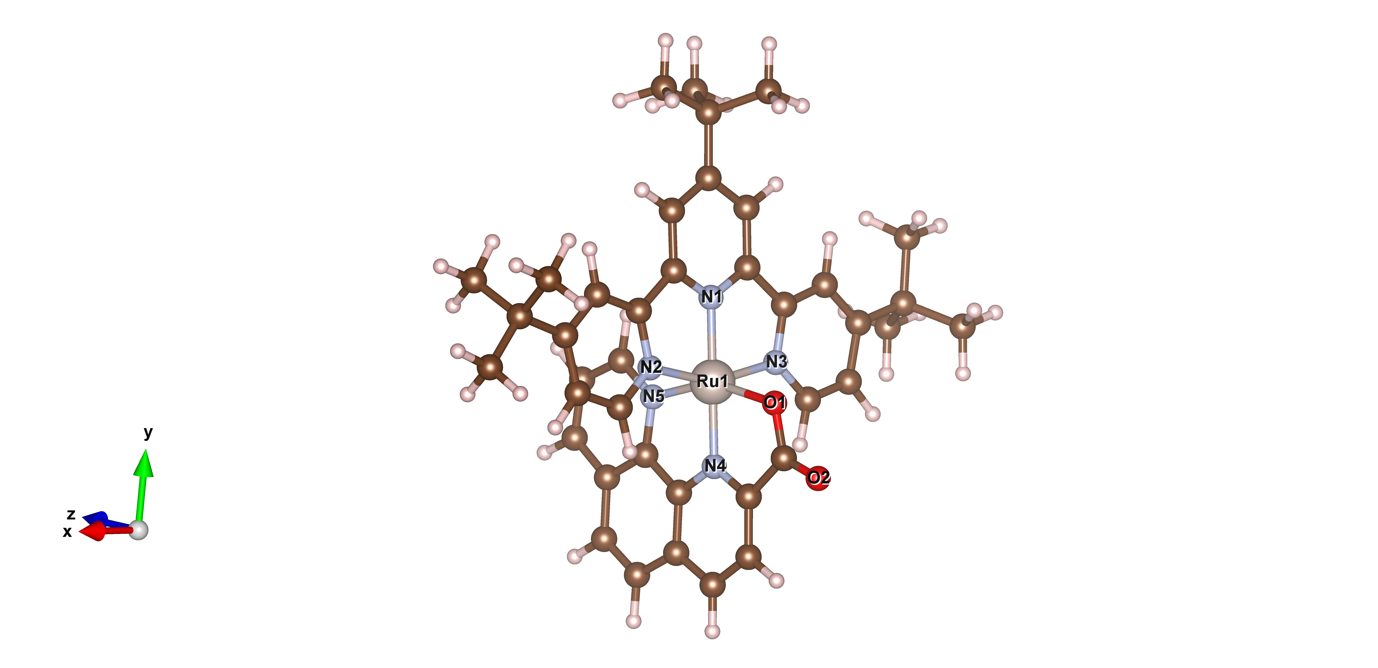 | 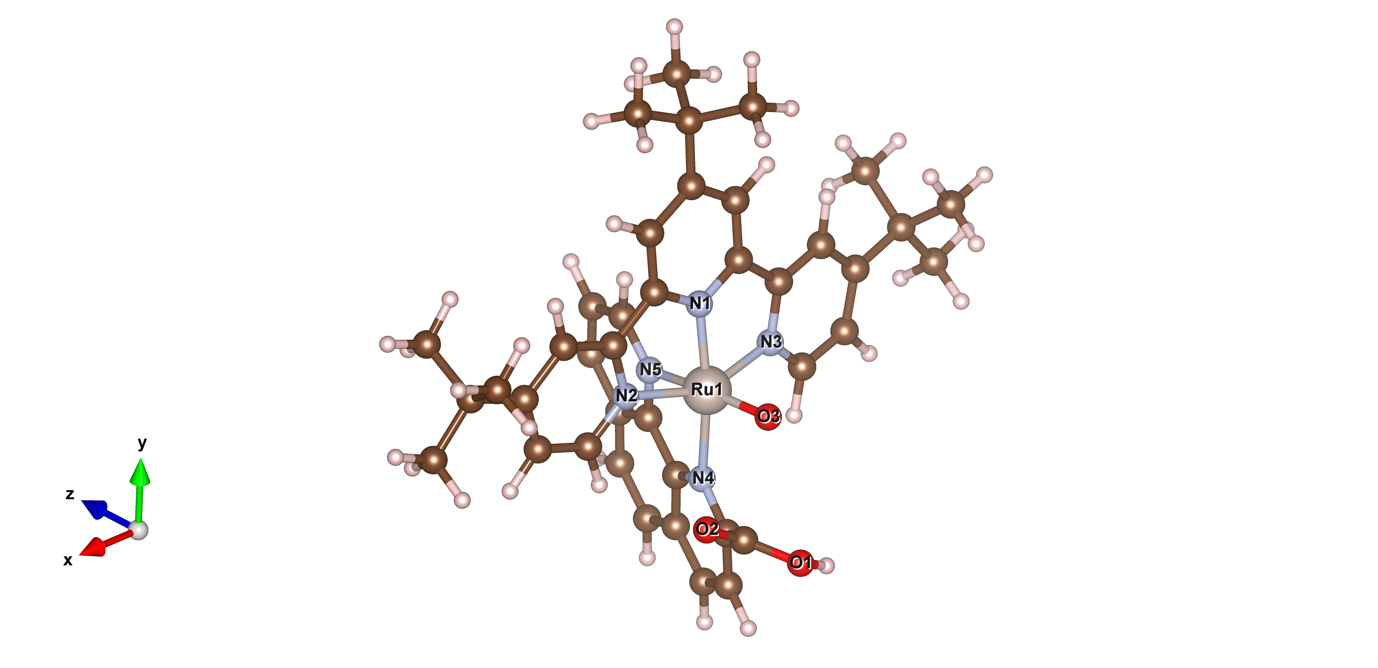 |
| --- | --- |
| **2** | **2** (Ru^V^=O) |
| 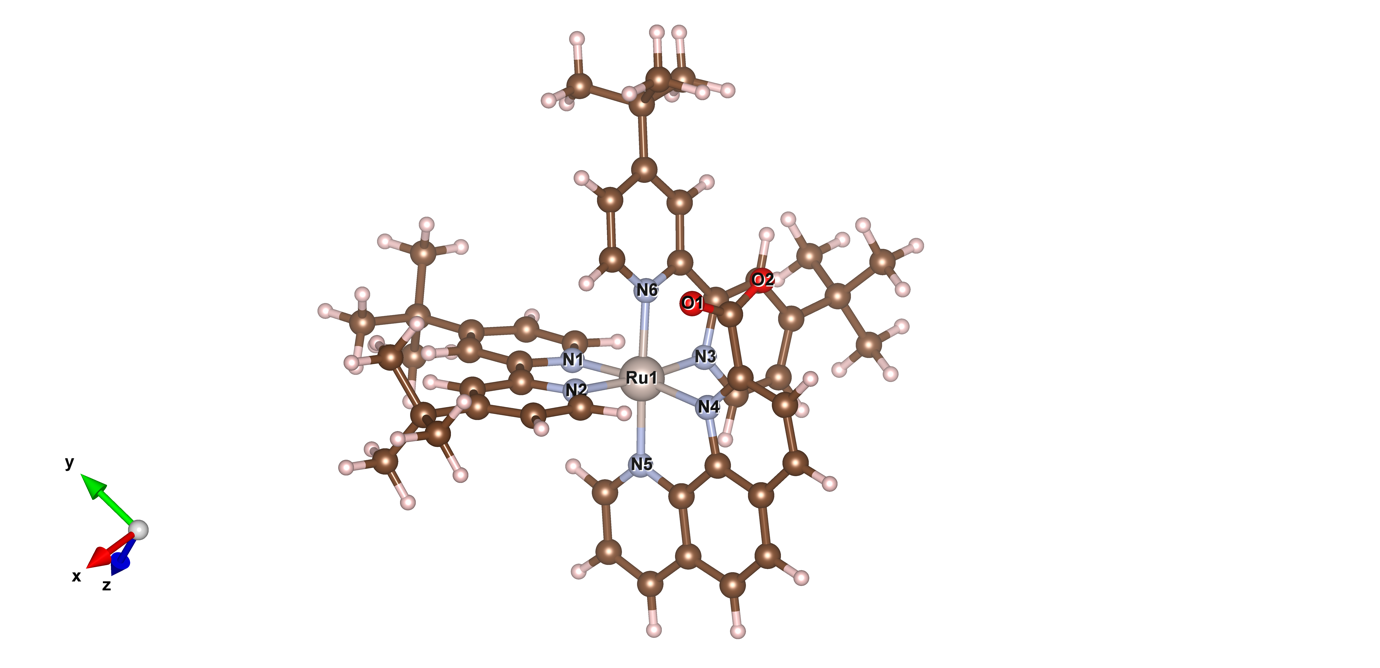 | 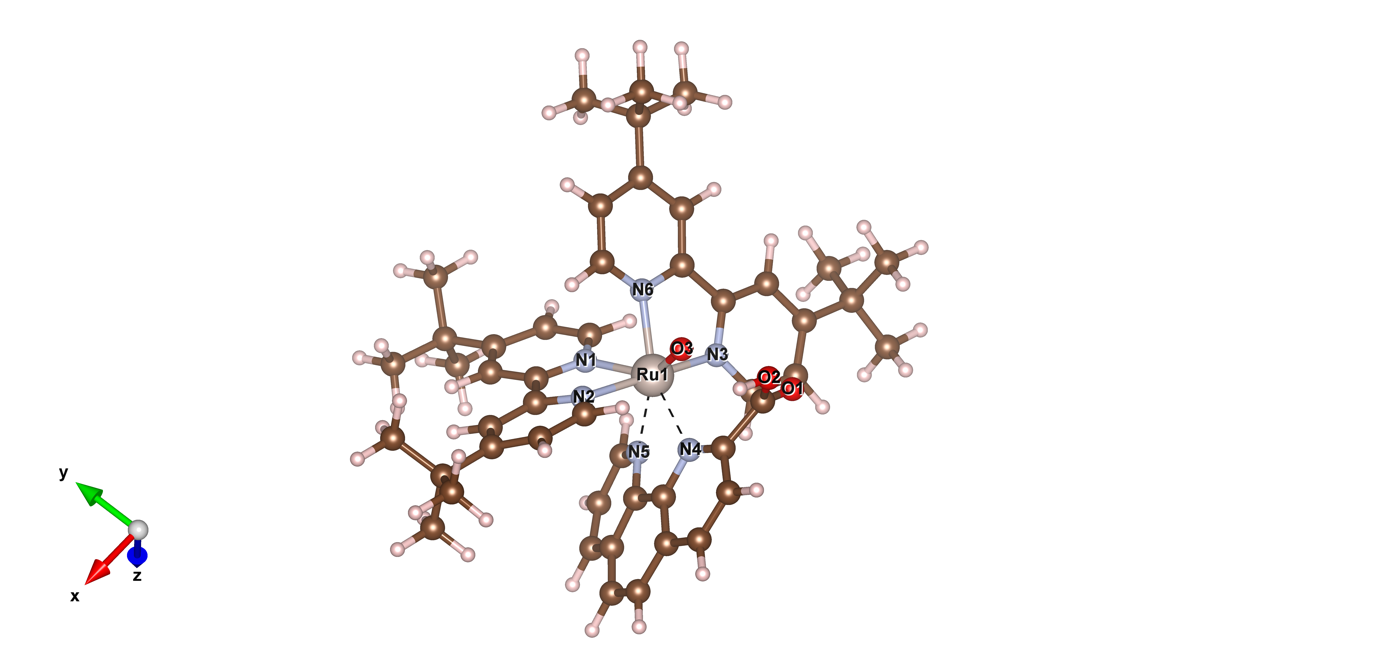 |
| **1** | **1** (Ru^V^=O) |

**Figure S17.** Optimized geometries of all the ruthenium complexes and corresponding Ru(V)Oxo species.

**The cartesian coordinates of the optimized geometries:**

**1_MeCN**

C 0.4450445924 4.4234131193 5.125108561

C 0.7090456135 4.7440901875 3.6312798462

C -0.3495861459 5.7563819099 3.1214662672

C 2.1049549856 5.3859212551 3.5041349888

C 0.586012913 3.4515374327 2.8174659777

C 1.6329644455 2.9311217497 2.0417780903

C 1.4712246919 1.7504773114 1.3018671917

N 0.2860512536 1.0626318308 1.3185859497

C -0.7334508087 1.5507478688 2.0650095214

C -0.6203100735 2.7179156646 2.812155999

Ru 0.1931750225 -0.6043328591 0.1195143607

N 2.1248526932 0.0196149926 -0.2182603886

C 3.0192494519 -0.5896612417 -1.0265657199

C 4.3206934462 -0.1171696634 -1.1990375726

C 4.7556417095 1.0363247033 -0.5201747552

C 6.1674735631 1.6177539817 -0.6562162714

C 6.0638510915 3.0652955279 -1.2024833603

C 6.8407095678 1.6346080569 0.7405937235

C 7.0461833613 0.7910093707 -1.6160032775

C 3.8112031403 1.6599540898 0.3167724939

C 2.5172035976 1.1492899327 0.4580564341

N -1.8093077386 -1.011928523 0.4115525315

C -2.3121828624 -1.9708528132 1.2201430345

C -3.6796646951 -2.2185399935 1.3393160642

C -4.6056587681 -1.4605990275 0.5984337646

C -6.1225921607 -1.6699815569 0.6702303655

C -6.5061920163 -2.8078340791 1.6369916035

C -6.7869029361 -0.3575516427 1.1610659354

C -6.6509094697 -2.0226966572 -0.7441973115

C -4.0667566635 -0.4690637403 -0.2420083593

C -2.6875677694 -0.2562307782 -0.3264862732

N 0.3264176437 -2.4037833235 -0.9806084709

C 0.7274923418 -3.4671352473 -0.1934018423

C 0.8517885853 -4.7964559132 -0.678433507

C 1.2905185802 -5.829526995 0.2186415444

C 1.6016182813 -5.5537542627 1.5296601299

C 0.5338794252 -5.0225037606 -2.0434155866

C 0.140148589 -3.9516598268 -2.8248643831

C 0.0501265018 -2.6417533674 -2.2797931141

C -0.3340496815 -1.5062646265 -3.2258532253

O 0.6421717047 -0.8910614212 -3.7428548465

O -1.5713544301 -1.3645986319 -3.4356395278

C 1.0468751124 -3.1848554751 1.1701303613

C 1.4915986907 -4.2172697941 2.0445109906

C 1.8025679748 -3.8558674543 3.3802395424

C 1.6620621372 -2.5295668156 3.7739374553

C 1.2072320176 -1.5677509074 2.8515415606

N 0.8973791342 -1.8807042837 1.5801721582

N -0.6894806695 0.6792290935 -1.2424301864

C -0.0282804553 1.5709340373 -2.0136405157

C -0.6808166404 2.5628224502 -2.7375720859

C -2.086395623 2.6753276315 -2.6921824589

C -2.807437022 3.7774936471 -3.4762341881

C -4.3377931331 3.7172681865 -3.2991875371

C -2.3058918971 5.1567800554 -2.9757423641

C -2.4781508801 3.6194365742 -4.9829957994

C -2.7583894545 1.7372612241 -1.8947968553

C -2.053498426 0.7536797857 -1.1870857331

H 0.5287254155 5.3445158252 5.7205773278

H 1.1800731142 3.6980207024 5.5040011473

H -0.5605060685 4.0093180151 5.2825797906

H -0.2718993526 6.6915164695 3.6955836547

H -1.3723565967 5.3717762848 3.2380062492

H -0.1873115036 5.9880191532 2.0584116715

H 2.1365869504 6.3042503943 4.1072021046

H 2.3340347284 5.6622556871 2.4642203639

H 2.8968856919 4.7171905946 3.8728471586

H 2.587427795 3.4455248728 1.9992443064

H -1.6531128753 0.9716177807 2.0520136839

H -1.4848381667 3.0458968058 3.385782548

H 2.6598894242 -1.4689565363 -1.5537658122

H 4.9766316395 -0.6652220035 -1.870191906

H 5.4876191964 3.7164636986 -0.5304108298

H 5.5791991121 3.0752081432 -2.1898963371

H 7.0716809198 3.4937356758 -1.3066860545

H 6.2817677318 2.2519916132 1.4576332319

H 7.8559108683 2.0498291035 0.6563239662

H 6.9159055429 0.6160390417 1.1490810348

H 6.6270588218 0.7659303841 -2.632656185

H 7.1731034024 -0.2431926638 -1.2633647903

H 8.0439525271 1.2480890975 -1.6771906706

H 4.0854943742 2.555878792 0.8674688978

H -1.5863450373 -2.5472756761 1.7880549606

H -3.9983108613 -3.0091970505 2.0135605718

H -6.1768564744 -2.5971905313 2.665236419

H -6.0780643679 -3.770916299 1.3220659494

H -7.5998745203 -2.9169037148 1.6522178085

H -6.4185483798 -0.0843218093 2.1610257957

H -7.8770272637 -0.4939942769 1.2189185308

H -6.5867025702 0.4812207102 0.4797405964

H -6.1812055222 -2.9448387901 -1.1169181803

H -6.4528145377 -1.2195791781 -1.4676548092

H -7.7389831222 -2.1797911779 -0.7033101592

H -4.7280015327 0.1397323202 -0.8526019144

H 1.3768316789 -6.8457370762 -0.1675207215

H 1.9378561852 -6.3451018664 2.1999047423

H 0.6075152888 -6.0275901611 -2.4599392173

H -0.1063919328 -4.0882778769 -3.8771595798

H 2.1477432446 -4.6163838148 4.0807082132

H 1.8949484358 -2.215189068 4.7901436178

H 1.0797297043 -0.5272134325 3.1379556058

H -4.6331574479 3.8545399449 -2.2482842387

H -4.7523000394 2.7637483187 -3.6586064439

H -4.8018848384 4.5244219317 -3.8835626468

H -2.5219355837 5.2847085174 -1.904520159

H -2.8148884647 5.9595111802 -3.5299097799

H -1.2236749158 5.2760166069 -3.1246342417

H -2.8156756192 2.640750707 -5.3546383499

H -1.4001757677 3.7071546821 -5.1771470205

H -2.9910040857 4.4048066701 -5.5580583834

H 1.0516855717 1.4658371172 -2.0454680743

H -0.0766333215 3.2443069171 -3.3334807612

H -3.8396680356 1.7691312563 -1.8075236116

**2_MeCN**

C -0.7309077056 5.8431215988 1.8062102227

C 0.0288903955 5.5487409816 0.4871184031

C -0.7040649658 6.2285398991 -0.698287565

C 1.4455851078 6.1477563721 0.5953268279

C 0.0546147722 4.0317258467 0.2516378507

C 1.2512202556 3.2998124234 0.1326056614

C 1.2191746956 1.9158179261 -0.0865969921

N 0.0234099054 1.2684443442 -0.1820746869

C -1.1617325357 1.9404032881 -0.0764810405

C -1.1606843048 3.3203063585 0.141501018

Ru 0.0005558953 -0.689512901 -0.4833662665

N 2.0371817135 -0.3176443526 -0.4530943106

C 3.0368699126 -1.2123182497 -0.5996127939

C 4.3850550042 -0.8549129505 -0.5413313355

C 4.7572089773 0.4850764645 -0.3233180723

C 6.2140647169 0.9563540445 -0.2470361077

C 6.4594887738 2.0147307945 -1.3533454609

C 6.464209383 1.5917990963 1.1452066808

C 7.2106275007 -0.2030515789 -0.4455350652

C 3.7052024177 1.4087257701 -0.1717091909

C 2.3684464641 1.0074013537 -0.2362112001

N -2.0275861507 -0.2731375807 -0.4344624854

C -2.329746922 1.0543850942 -0.2156585619

C -3.6601691855 1.4879037025 -0.1374152057

C -4.7282524394 0.5878830516 -0.2777734456

C -6.1982242921 1.0148092648 -0.1992308881

C -6.8811482365 0.2483828918 0.9630483848

C -6.3557363833 2.528125676 0.0498607127

C -6.8951061676 0.6582365789 -1.5378804251

C -4.3834570434 -0.7632849245 -0.5006005588

C -3.048610841 -1.1501473902 -0.5719965439

N -0.0196862691 -2.6446344459 -0.7889543937

C -0.0212563017 -3.4409616223 0.3143854914

C -0.0343631987 -4.8525805737 0.2258378467

C -0.0350889583 -5.5764624921 1.4713766535

C -0.0238974862 -4.9113581921 2.6806854973

C -0.0454550019 -5.4053825417 -1.0873074502

C -0.0430738871 -4.5625633883 -2.2035297109

C -0.0298036869 -3.1614973481 -2.0316896401

C -0.0242284791 -2.0802402388 -3.092395623

O -0.0105223249 -0.8513011267 -2.6221472382

O -0.0322787138 -2.3699191502 -4.30394063

C -0.0093436173 -2.7395670046 1.551090578

C -0.0110729744 -3.4691936895 2.7663770386

C 0.0000606233 -2.7167981374 3.9689620441

C 0.0116775766 -1.3251581122 3.8931041719

C 0.0126500568 -0.673899128 2.6419100542

N 0.0024951909 -1.3553737582 1.4814117311

H -0.7499593944 6.9279642466 1.9879300601

H -0.2334918983 5.3577395153 2.6589364163

H -1.7710092735 5.4900927693 1.7684815467

H -0.7308087824 7.317223265 -0.5420187563

H -1.7404216873 5.8758161526 -0.7952870904

H -0.1826196362 6.0269122594 -1.6456148642

H 1.3674440701 7.2306959651 0.7663346669

H 2.0247573475 5.9974585382 -0.3277701105

H 2.0076129402 5.716875188 1.437210948

H 2.2117042432 3.8001748048 0.2066770106

H -2.1088886845 3.8452864708 0.223722

H 2.7323950398 -2.2432636178 -0.7689135793

H 5.1267454879 -1.6388059043 -0.6700067579

H 5.8112335108 2.8934812514 -1.2303881044

H 6.2747820913 1.5873390605 -2.349853639

H 7.5041407502 2.3568952627 -1.311167557

H 5.8148037152 2.4607517011 1.3211978831

H 7.5085315055 1.9308154598 1.2138558985

H 6.2844125805 0.8595598331 1.946154729

H 7.0920320484 -0.6778097436 -1.4307138774

H 7.0992408657 -0.9738241118 0.331286081

H 8.2363140927 0.1872253603 -0.3840064441

H 3.9230345957 2.460186644 -0.0009030508

H -3.848818604 2.5431551181 0.035118252

H -6.3950936579 0.4810346708 1.9220480397

H -6.8440891544 -0.8395459263 0.8131579054

H -7.9384056717 0.5449825237 1.029757526

H -5.8995042188 2.8345597463 1.0029280889

H -7.4251930118 2.7772832774 0.0970931345

H -5.9101501845 3.1235693918 -0.76073623

H -6.8607443559 -0.4209606384 -1.7418969623

H -6.4171919611 1.1831881851 -2.3780997679

H -7.951704507 0.9615586334 -1.4958364413

H -5.1468855235 -1.5291551356 -0.6219287425

H -0.0447365934 -6.6663723833 1.447695663

H -0.0247740967 -5.4793047631 3.6114392312

H -0.0557288894 -6.4873580877 -1.2171868321

H -0.0512502385 -4.968867576 -3.2136322745

H -0.0006981717 -3.2239308869 4.9336720082

H 0.0202646176 -0.7172774434 4.7964920372

H 0.0219957509 0.4113988231 2.5646867021

H -2.7660073222 -2.186653843 -0.7441667269

**1(V)-Oxo**

C 2.5957432534 3.0904566828 4.8426899697

C 3.0623882949 3.3496435187 3.3857005663

C 2.8080442767 4.8337135454 3.0087027235

C 4.5736553358 3.0619533561 3.289900101

C 2.2421722208 2.4671997434 2.4449895895

C 2.8250046528 1.5514248704 1.5555995188

C 2.0361868292 0.7584322928 0.7106577669

N 0.6811114834 0.855069123 0.7496316189

C 0.090520049 1.7472274438 1.5748536128

C 0.8325035817 2.5556548752 2.4241417926

Ru -0.393698276 -0.4708032162 -0.6103031995

N 1.6451660401 -0.6933046613 -1.1453789398

C 2.0573074561 -1.466140084 -2.1699851562

C 3.3970094921 -1.8044126371 -2.3511914155

C 4.3731271942 -1.3517368045 -1.4447865114

C 5.8485174992 -1.7449146608 -1.5284624107

C 6.7150065861 -0.4618679729 -1.6213619417

C 6.2101214407 -2.530748564 -0.2392093937

C 6.1431693447 -2.6325214095 -2.7535889351

C 3.9243988321 -0.502939056 -0.4137057933

C 2.5741375246 -0.1735500136 -0.2881863396

N -2.3215056963 -0.2035064578 0.0908761173

C -3.0203337993 -1.1490128755 0.7510401634

C -4.3325794688 -0.9376877037 1.1675573614

C -4.9801038195 0.2797291998 0.8890304399

C -6.4243803998 0.5765570157 1.2943717989

C -7.0608099023 -0.5893243276 2.076366764

C -6.4457412759 1.8467523302 2.1853822679

C -7.2532988529 0.8265367635 0.0059626696

C -4.229276445 1.2488230458 0.1912876146

C -2.9139006328 1.000974154 -0.1985773874

N -0.4726783295 -2.7238245404 -0.9333464622

C 0.3638612128 -3.4348211523 -0.1382475455

C 0.8280348033 -4.7412513594 -0.4577445075

C 1.6932510367 -5.4086948121 0.4759191309

C 1.9828672763 -4.8429565686 1.6947646527

C 0.3619486957 -5.2980685955 -1.6716236107

C -0.5642053537 -4.5940471858 -2.4349376921

C -0.9795973161 -3.3025654576 -2.032491407

C -2.1692334398 -2.6561154356 -2.7259464168

O -3.1735132834 -2.3631554041 -2.1022232595

O -2.1416916267 -2.5796162801 -4.0727674666

C 0.6828298084 -2.8378688436 1.1138911371

C 1.4404055773 -3.5631970647 2.0717750319

C 1.5512319557 -2.9989220465 3.366626163

C 0.867642673 -1.8222629089 3.6495712193

C 0.1649474655 -1.1561199364 2.6260686213

N 0.1166336362 -1.6230727191 1.3705419885

N -0.7904841708 1.5508869819 -1.1582699229

C 0.0804085024 2.3335693829 -1.8207593621

C -0.2841478038 3.6037724351 -2.2522126363

C -1.585299591 4.0957529914 -2.0072442543

C -1.9841330758 5.49653867 -2.4753016793

C -3.4341028168 5.8528539015 -2.092079733

C -1.0270324182 6.5278713163 -1.8212908009

C -1.8452774503 5.5576021992 -4.0195372942

C -2.468695288 3.2401669134 -1.3284944378

C -2.062857119 1.9639279747 -0.9146574088

H 3.1879759506 3.7144988981 5.5269389306

H 2.7427979219 2.0365104654 5.1193796865

H 1.5367397684 3.343034491 4.9882169018

H 3.3905309934 5.4817479058 3.6790250134

H 1.7486780867 5.1066323639 3.10910005

H 3.1215157521 5.0327792979 1.9737385941

H 5.1069443541 3.7117330224 3.9969372189

H 4.9680629631 3.2722677328 2.285109618

H 4.8087391937 2.0198524304 3.5523886825

H 3.9033650695 1.4533958269 1.4993447043

H -0.9940794877 1.7963045502 1.5614900683

H 0.2926027077 3.2473205506 3.066000267

H 1.2880276349 -1.8224062468 -2.8481352974

H 3.6507401995 -2.435235333 -3.1981668613

H 6.597509854 0.1832092365 -0.7398152042

H 6.4535334389 0.1219693814 -2.5155348608

H 7.7748063383 -0.7456667464 -1.6902122093

H 6.0631424517 -1.9235717669 0.6648051643

H 7.267748514 -2.8282852456 -0.2801635717

H 5.5965317663 -3.4389851931 -0.1495986483

H 5.9120869873 -2.1145007911 -3.695917309

H 5.5780833451 -3.57542264 -2.7207819249

H 7.2121404519 -2.8855815511 -2.7647050814

H 4.6325590359 -0.1013105329 0.304538398

H -2.5101399465 -2.0879936664 0.9380957958

H -4.8313746467 -1.7429389726 1.6986136561

H -6.5164684149 -0.8005933938 3.0083494908

H -7.1040986046 -1.5093496361 1.4754806615

H -8.0911961317 -0.31951841 2.345542843

H -5.8425313967 1.6987748784 3.0927333145

H -7.4813525711 2.0574293176 2.4880209582

H -6.0646894405 2.7303937306 1.6553415528

H -7.2408778835 -0.0598513378 -0.6443236441

H -6.8727746479 1.6824003732 -0.568130127

H -8.2956630617 1.0402751022 0.2827255006

H -4.6752315254 2.2085169615 -0.0514180279

H 2.081180571 -6.3923689314 0.2145858527

H 2.5976540134 -5.376699187 2.4186357325

H 0.6955178174 -6.2871357883 -1.9829434181

H -1.0005992633 -5.0428187089 -3.3252220024

H 2.1341030576 -3.5065007388 4.1344404294

H 0.8746037554 -1.3854282734 4.6460786483

H -0.3478848534 -0.2243675877 2.8382088248

H -3.5830821421 5.8437798676 -1.0022500145

H -4.1605205353 5.1687291663 -2.5549672267

H -3.6597247783 6.8667522764 -2.4497637652

H -1.0934240002 6.4826900652 -0.7245330748

H -1.3100218621 7.5397355324 -2.1447340714

H 0.0190241154 6.360769402 -2.112310097

H -2.5077310275 4.8236025254 -4.5006564767

H -0.8152184945 5.3612590298 -4.3469554602

H -2.1267702491 6.5612389797 -4.3689672263

H 1.0736624708 1.9268799966 -1.9925605372

H 0.4602544589 4.1981091417 -2.7762875889

H -3.4847622616 3.5568609047 -1.1200693827

O -0.8507349704 -0.6064831026 -2.2976727906

H -1.2492987611 -2.7589304148 -4.4326118088

**2(V)-Oxo**

C 0.0945752508 4.125621372 0.0248813762

C 1.2999475563 3.389018432 0.0245434113

C 1.2763751363 2.0043428384 -0.1529797747

N 0.0864673079 1.3788419109 -0.3464065656

C -1.1101031778 2.0295328425 -0.3009443023

C -1.1178624242 3.4119059362 -0.1201155297

Ru 0.0998926828 -0.5827120145 -0.6978937016

N 2.0567643139 -0.2679477019 -0.1762388997

C 3.0000443703 -1.2256669112 -0.0335148112

C 4.3412755127 -0.8989096588 0.1472282174

C 4.7512110852 0.4478784482 0.2036361939

C 3.7375201674 1.4294462239 0.0836210262

C 2.4096962373 1.0686474777 -0.0916838783

N -1.9566240124 -0.2089585767 -0.5094143374

C -2.2661644963 1.127566192 -0.3906908911

C -3.5960398534 1.5309843494 -0.3070474955

C -4.6471928621 0.5886443829 -0.3274479609

C -4.279160646 -0.7649981277 -0.4385259333

C -2.9346918327 -1.1303707198 -0.5298021737

N 0.1298160078 -2.7494150384 -0.6806842482

C -0.2721920828 -3.2876247897 0.5124133816

C -0.4888739151 -4.684547144 0.6893586239

C -0.8985621953 -5.1703817512 1.9777399116

C -1.065264233 -4.3122887869 3.0370581948

C -0.279858498 -5.5188160838 -0.4336720802

C 0.1548534077 -4.9625030636 -1.6298127083

C 0.3695871081 -3.5700641621 -1.7229981832

C 1.0532348688 -3.0047649301 -2.9515837649

O 0.4949179166 -3.2714348605 -4.1464560968

O 2.1163026177 -2.4154112115 -2.8647934569

C -0.4517230538 -2.3967330711 1.6163880421

C -0.8386270714 -2.9005073125 2.8880796623

C -0.9717200089 -1.9647630404 3.944633109

C -0.7164465513 -0.618438948 3.7073548345

C -0.3501047313 -0.1973456473 2.4162509961

N -0.2208289064 -1.0662930711 1.4024055522

H 2.2515988169 3.8876006788 0.1749020561

H -2.0693020501 3.9334441963 -0.080691246

H 2.6656105164 -2.2557082497 -0.0908472958

H 5.0512860387 -1.7154242099 0.2366047584

H 3.9870165958 2.4850397049 0.1338073001

H -3.8108784124 2.5915225234 -0.2131108299

H -5.0195285691 -1.5585413145 -0.4737804455

H -1.0676714841 -6.2396561592 2.0968364008

H -1.3667178444 -4.689072269 4.0134376316

H -0.4428753638 -6.5923203926 -0.3511304733

H 0.3602254767 -5.593094845 -2.4926304869

H -1.2694563612 -2.3081012602 4.9347054917

H -0.7974203761 0.1222422601 4.4999543534

H -0.1560475724 0.8497721354 2.2020244094

H -2.6342154907 -2.1682570113 -0.6427847436

O -0.0842547565 -0.6037268308 -2.4070186419

H -0.3899064156 -3.6810097098 -4.0517190201

C -6.0976724679 1.0590679571 -0.2267185818

C -6.2765136542 1.8278074192 1.1102023196

H -5.6273039905 2.7120771874 1.1693141468

H -7.3183244464 2.1685457868 1.1915849049

H -6.0561663872 1.1768358387 1.9682191527

C -6.3929500621 2.0063333803 -1.4209323244

H -6.2488686675 1.4854546697 -2.3783360877

H -7.4377974749 2.3427129585 -1.3622159932

H -5.7506393353 2.897570388 -1.4101733429

C -7.0939048976 -0.1163070696 -0.2632990811

H -7.0286637643 -0.678044931 -1.2064186446

H -6.9369376331 -0.8106378382 0.5748552721

H -8.1155091449 0.2786714108 -0.1814148218

C 0.0623883973 5.6434322513 0.2026629566

C 1.4721678849 6.2457273036 0.3627123606

H 1.9833818973 5.8597880954 1.2566615385

H 2.1019395492 6.0559906776 -0.5185556103

H 1.3825869853 7.3342214519 0.4771774763

C -0.6105677238 6.266551284 -1.0512122699

H -1.6427705766 5.9151489436 -1.1842298945

H -0.6378747752 7.3591378541 -0.9354052626

H -0.0413515083 6.0268831688 -1.9604794115

C -0.7715536159 5.97948458 1.4687567083

H -1.8119621181 5.6379894729 1.383836797

H -0.3259874703 5.5216187766 2.3632524514

H -0.7859584699 7.0696891457 1.6066198625

C 6.2018751098 0.8746581549 0.395777742

C 6.6210865909 1.7441169359 -0.8226031286

H 6.0126047999 2.6541011975 -0.9122161789

H 7.6690916775 2.049650253 -0.6948272331

H 6.5353870519 1.1733623857 -1.7579036997

C 6.3021196772 1.7160228202 1.698379844

H 5.9913257404 1.1236585117 2.5704837844

H 7.3468491561 2.0256198677 1.8414508629

H 5.6838976551 2.6228757498 1.6552357594

C 7.1576998624 -0.3291561482 0.5029062159

H 6.920983202 -0.9633886901 1.3693001511

H 7.1388963103 -0.9472076758 -0.4062694081

H 8.1834858928 0.0411214443 0.6324517096


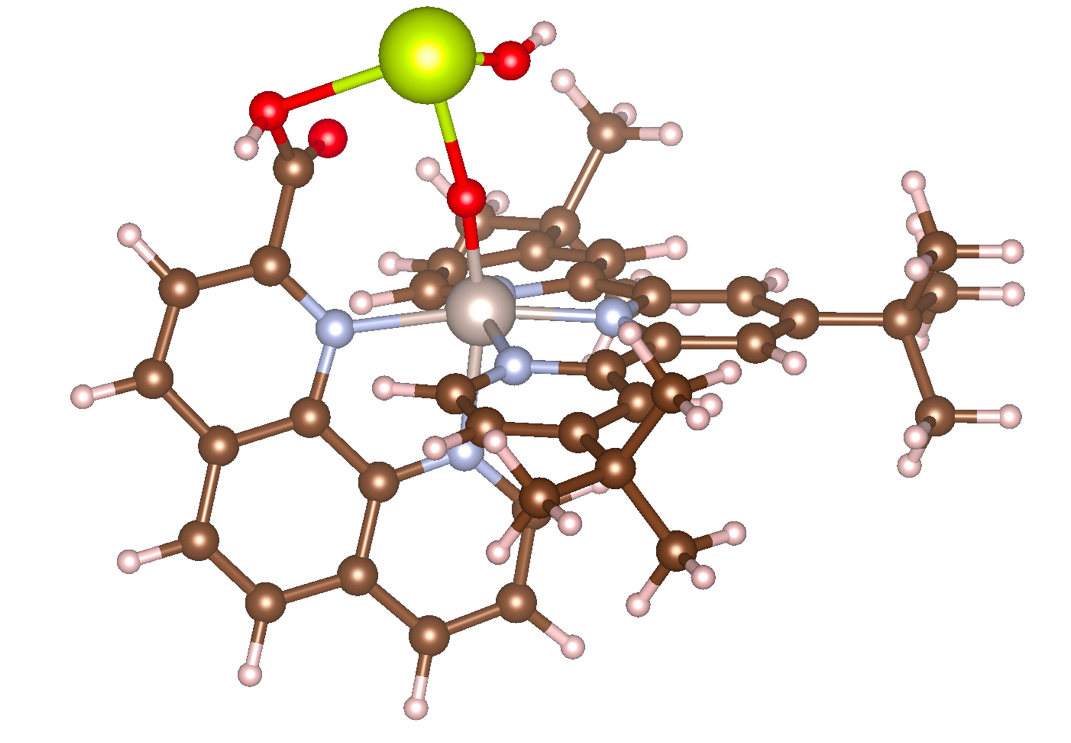


**Figure S18**: Ru(IV)-O-Ce(OH) structural model employed to assess the I2M-HC pathway. C, O, H, N, and Ce are represented by brown, red, pinkish-white, blue, and green spheres, respectively.


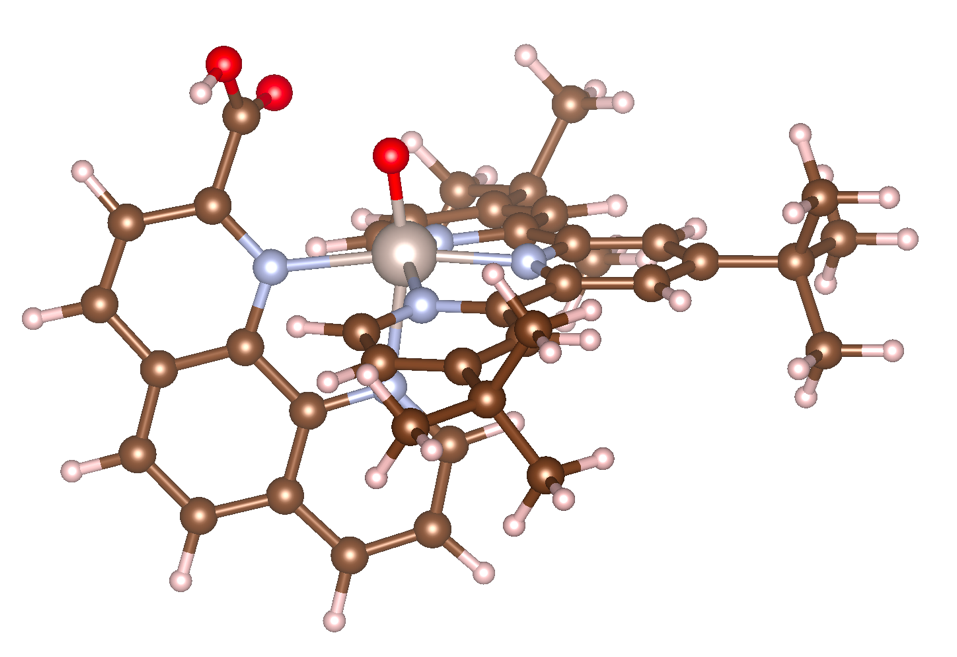


**Figure S19**: Ru(IV)-oxyl DFT structural model employed to assess the relative stability of Ru(IV)-oxyl and Ru(V)-oxo.

**6. References:**

1. S Stoll, A Schweiger, J. Magn. Reson., 2006, 178, 42-55.

2. G. Sheldrick, Acta Cryst., 2008, A64, 112-122.

3. A.L.Spek, J. Appl. Cryst., 2003, 36, 7-11.

4. [B. P. Sullivan](https://pubs.acs.org/action/doSearch?field1=Contrib&text1=B.+P.++Sullivan), [D. J. Salmon](https://pubs.acs.org/action/doSearch?field1=Contrib&text1=D.+J.++Salmon), [T. J. Meyer](https://pubs.acs.org/action/doSearch?field1=Contrib&text1=T.+J.++Meyer) Inorg. Chem. 1978, 17, 12, 3334–3341.

5. [B Das](https://pubs.rsc.org/en/results?searchtext=Author%3ABiswanath%20Das),   [L Ezzedinloo](https://pubs.rsc.org/en/results?searchtext=Author%3ALida%20Ezzedinloo),   [M Bhadbhade](https://pubs.rsc.org/en/results?searchtext=Author%3AMohan%20Bhadbhade),   [M P. Bucknall](https://pubs.rsc.org/en/results?searchtext=Author%3AMartin%20P.%20Bucknall) and  [S B. Colbran](https://pubs.rsc.org/en/results?searchtext=Author%3AStephen%20B.%20Colbran) Chem. Commun., 2017,53, 10006-10009.

6. M. J. Frisch, G. W. Trucks, H. B. Schlegel, G. E. Scuseria, M. A. Robb, J. R. Cheeseman, G. Scalmani, V. Barone, G. A. Petersson, H. Nakatsuji, X. Li, M. Caricato, A. V. Marenich, J. Bloino, B. G. Janesko, R. Gomperts, B. Mennucci, H. P. Hratchian, J. V. Ortiz, A. F. Izmaylov, J. L. Sonnenberg, Williams, F. Ding, F. Lipparini, F. Egidi, J. Goings, B. Peng, A. Petrone, T. Henderson, D. Ranasinghe, V. G. Zakrzewski, J. Gao, N. Rega, G. Zheng, W. Liang, M. Hada, M. Ehara, K. Toyota, R. Fukuda, J. Hasegawa, M. Ishida, T. Nakajima, Y. Honda, O. Kitao, H. Nakai, T. Vreven, K. Throssell, J. A. Montgomery Jr., J. E. Peralta, F. Ogliaro, M. J. Bearpark, J. J. Heyd, E. N. Brothers, K. N. Kudin, V. N. Staroverov, T. A. Keith, R. Kobayashi, J. Normand, K. Raghavachari, A. P. Rendell, J. C. Burant, S. S. Iyengar, J. Tomasi, M. Cossi, J. M. Millam, M. Klene, C. Adamo, R. Cammi, J. W. Ochterski, R. L. Martin, K. Morokuma, O. Farkas, J. B. Foresman, D. J. Fox, Wallingford, CT, 2016.

7. S. Grimme, S. Ehrlich, L. Goerigk, Journal of computational chemistry 2011, 32, 1456-1465.

8. A. V. Marenich, C. J. Cramer, D. G. Truhlar, The Journal of Physical Chemistry B 2009, 113, 6378-6396.
